# Research Progression of the Genus *Merremia*: A Comprehensive Review on the Nutritional Value, Ethnomedicinal Uses, Phytochemistry, Pharmacology, and Toxicity

**DOI:** 10.3390/plants10102070

**Published:** 2021-09-30

**Authors:** Tomi Lois Olatunji, Ademola Emmanuel Adetunji, Chijioke Olisah, Oladayo Amed Idris, Oluwaseyi Damilare Saliu, Frances Siebert

**Affiliations:** 1 Unit for Environmental Sciences and Management (UESM), Faculty of Natural and Agricultural Sciences, North-West University, Potchefstroom 2520, South Africa; dayoamed@yahoo.com (O.A.I.); frances.siebert@nwu.ac.za (F.S.); 2 School of Life Sciences, University of KwaZulu-Natal, Durban 4001, South Africa; adetunjiademola@hotmail.com; 3 Department of Botany, Institute for Coastal and Marine Research, Nelson Mandela University, Port Elizabeth 6031, South Africa; olisah.chijioke@gmail.com; 4 Department of Chemical Sciences, University of Johannesburg, Johannesburg 2028, South Africa; oluwaseyi229@gmail.com

**Keywords:** bibliometric, Convolvulaceae, fodder, *Merremia*, merremins, resin glycosides

## Abstract

The genus *Merremia* Dennst. ex Endl. (Convolvulaceae) is a rich source of structurally diverse phytochemicals with therapeutic relevance. This review presents the first comprehensive, up-to-date information and research progression on the nutritional value, ethnomedicinal uses, phytochemistry, pharmacological activities, and toxicity of the genus *Merremia*. Using the key search term “*Merremia*”, relevant documents and information were retrieved from electronic databases. Relevant documents were uploaded in RStudio with installed bibliometric software packages and used for data retrieval, tabulation, and network visualization. Bibliometric analysis revealed that ca. 55% of the studies related to *Merremia* were published in the last decade, which can be grouped into four thematic areas: (i) drug formulation, (ii) taxonomy, (iii) chemical analysis, and (iv) treatment of diseases. Ethnomedicinal uses, phytochemistry, and biological activities studies showed that species in the genus are promising medicinal plants with various pharmaceutical potentials. However, clinical studies to validate the efficacy of the reported bioactivities and the mechanisms underlying the various activities are lacking and should constitute a future research focus. Additionally, reports on the nutritional and antinutritional constituents of *Merremia* species revealed that the species meet high nutritional quality criteria for animals and are therefore suitable for inclusion in livestock diets. The few available investigations on toxicity indicated that most *Merremia* species are safe for human and animal use but not with prolonged chronic administration.

## 1. Introduction

Natural medicines, whether from standardized plant extracts or pure compounds from plants, are valuable sources of new drugs in the pharmaceutical industries because of their diverse biologically active phytochemicals [1,2]. Over the years, medicinal plants have been used in many human populations all over the world [3] in the treatment of different ailments and diseases. This is largely due to their health benefits, effectiveness [4], affordability, and little or no side effects when compared to synthetic drugs that are expensive and may have adverse effects [5].

A large proportion of species used in traditional medicine globally are forbs (syn. herbs), which are one of the most species-rich plant life forms, especially in open ecosystems worldwide [6,7]. Several indigenous rangeland forb species are consumed as vegetables, thus forming a part of local people’s diet. They are economically useful crops that had bolstered food security historically when the main crops failed, and thus should be increased in production to solve the global food crises [8]. Forbs are important nutritive dietary components of several browsers as components of mixed pastures and have been reported to contain higher levels of phosphorus and crude protein, and lower fiber levels than shrubs or grasses [6]. Additionally, many of the forb species are used in traditional medicine to treat different ailments, and the broad range of bioactive phytochemicals present in some forb species can be potentially employed for the treatment and prevention of several diseases in modern medicine. Of these forbs, species in the genus *Merremia* have been the focus of several pharmacological and phytochemical studies. 

*Merremia* Dennst. ex Endl. is a large genus of over 100 species of flowering plants in the Convolvulaceae (morning glory) family [9]. Members of the family Convolvulaceae are usually recognized by their colorful funnel-shaped flowers [10,11]. Resin glycosides [12,13], calystegines [14], and tropane alkaloids [15] are considered the chemotaxonomic markers of the Convolvulaceae family.

The genus *Merremia*, commonly referred to as wood roses, is distributed in tropical and sub-tropical regions all over the world [16]. Several species in the genus are of reputed medicinal value and are used in traditional medicine across continents. For example, in Sri Lanka, the whole plant of *M. emarginata* is prepared as a decoction and used in the traditional treatment of diabetes [17]. In India, leaves of *M. hederacea* have been reported to be used in treating chapped hands and feet [18]. In the Philippines, the leaves of *M. peltata* are used for treating skin sores, inflammation, and stomach pain [19]. The leaves and rhizomes of *M. vitifolia* are used in the traditional treatment of eye inflammation, dysentery, urinary diseases, and jaundice in Bangladesh [20]. It is also evident from the literature that the genus *Merremia* is a rich source of biologically active phytochemicals [21,22,23] since extracts from the different species have shown pharmacological activities in several in vitro and in vivo studies. Thus, species in the genus *Merremia* would be promising alternative sources of new pharmaceutical leads in treating several diseases and ailments. Despite the widespread ethnopharmacological importance and different scientific investigations of phytochemistry and pharmacological activities, there is no comprehensive review analyzing, documenting, and revealing the pharmacological importance of this genus. 

Bibliometric analysis has been recognized as a useful statistical method that can qualitatively and quantitatively evaluate the trend in research efforts in a given area of interest [24,25]. Bibliometrics can thus be employed to assess both national and international research focus and is particularly useful in providing insights for future research [26]. 

This review presents the first comprehensive, up-to-date information and research progression on the nutritional value, ethnomedicinal uses, phytochemistry, and pharmacological activities of the species in the genus *Merremia*. Hence, this review is aimed to (i) identify the international research focus of the genus, (ii) provide insight into the emerging pharmacological potentials of *Merremia* species, (iii) highlight biological activities of extracts and isolated phytochemicals, (iv) highlight its potentials as livestock forage and (v) provide the scientific basis for further research on the nutritional value, pharmacological potentials and their mainstream application.

## 2. Materials and Methods

For the global bibliometric analysis of the genus *Merremia*, document retrieval and statistical analysis were conducted based on the method described in [27,28]. In summary, document information related to *Merremia* (between 1990–2020) was retrieved from 10 databases indexed in the Web of Science (WoS) (“SCI EXPANDED, SSCI, A&HCI, CPCI-S, CPCI-SSH, BKCI-S, BKCI-SSH, ESCI, CCR EXPANDED, and IC”). The term “*Merremia*” was inputted as a search term in the topic module on the WoS database hosted in Clarivate Analytics as well as in the Scopus topical search engine. Only document types including “Article”, “Review,” “Editorial Material”, and “Book Chapter” were searched. Other document types such as “Meeting Abstract”, “Proceeding Paper”, and “Notes” were excluded. A total of 194 documents satisfied the search criteria in the WoS database. The total search yielded a total of 261 publications from the Scopus database. It should be noted that the documents excluded from both databases were because they were either pre-publication data that may have been published as an article or post-article data taken from the primary articles [26,27]. The identified number of articles from both databases were downloaded in Bibtex and uploaded in RStudio for further statistical processing. 

RStudio Inc. (Version 1.1.463—© 2021–2018, Boston, MA, USA) with installed bibliometric software packages was used for data retrieval, tabulation, and network visualization. Duplicate records from the hybrid databases were merged using R codes, and these were taken as a single article. A total number of 357 publications were used for bibliometric analysis. Commands for analyzing bibliometric indicators were retrieved from https://cran.r-project.org/web/packages/bibliometrix/vignettes/bibliometrix-vignette.html, accessed on 15 February 2021.

Keyword network map was visualized using VOS viewer (Nees Jan van Eck and Ludo Waltman; Leiden; The Netherlands, version 1.6.15—© 2021–2020).

Information on the nutritional value, ethnomedicinal uses, phytochemistry, biological activities, and toxicity studies were retrieved from relevant articles, books, webpages and other online materials searched using different databases. The search period was from inception to July 2021 in the different databases. The Boolean string used was “*Merremia*”, searching the “title”, “abstract”, “authors keyword”, and “keyword plus” of the documents. The search was limited to materials available in English. The relevant materials retrieved were used to present a comprehensive and up-to-date review write-up on the subject matter. All species names and synonyms were confirmed from the “World Flora Online (WFO)”. All chemical structures were drawn with Chem Draw.

## 3. Results 

### 3.1. Bibliometric Overview of the Genus—Merremia

A total of 357 documents with an average citation per article of 10.39 was retrieved from the merged databases (WoS and Scopus) (Table 1). Of all the documents present, 341 were research articles and 12 review articles. Other document types included book chapters and editorial materials, which revealed two documents in each. Documents were obtained from 188 journals and included 1005 author keywords and 2727 keywords plus. There was a slightly steady increase in documents produced between 1990 and 2020 with a major fluctuation in the first (1990–2000) and last (2011–2020) decades of the survey period. Peak numbers were recorded in 2009 with 33 articles, followed by 2020 and 2010 with 26 and 25 articles, respectively. Approximately 55% of the articles published on studies related to *Merremia* were published in the last decade (2011–2020). A high number of articles in these years may be due to the availability of funds, the advent of research ideas, and the emergence of sophisticated analytical tools for chemical analysis. 

### 3.2. Most Productive Countries 

To avoid multiple representations of authors, authors from the same country in an article were computed as one. We prioritized this bibliometric tool to recognize the most prolific countries on *Merremia* related research. As shown in Table 2, authors’ countries were ranked based on the number of articles and citations accumulated over the survey period. Brazil had the highest number of articles (*n* = 116), with 32.5% of the total number of articles. This was followed by India (*n* = 51, 14.3%), Japan (*n* = 27, 7.6%), USA (*n* = 26, 7.3%), and China (*n* = 18, 5.0%). Based on continental production, Asia and America were more prolific, having seven countries each in the top 20 authors’ countries on studies related to the genus *Merremia.* Most of the *Merremia* species are found in the Asian continent, which may be responsible for their high number of articles (https://www.cabi.org/isc/datasheet/33477, accessed on 23 March 2021). As for the Americas, the United States Department of Agriculture (USDA) plants database reported that seven species of *Merremia* occur in North America. Four of these species remain unique to the North American region, while three have been neutralized in other parts of the world. These species include *M. quinquefolia*, *M. cissoids*, and *M. umbellata* that are native to Florida, while *M. dissecta* (Alamo vine) occurs in Texas, Pennsylvania, and states in the south-eastern region (https://www.wildflower.org/expert/show.php?id=7618, accessed on 28 March 2021). A country that hosts a substantial number of studied species is expected to attract more scientific research compared to other countries [29], which explains why the USA is ranked fourth in the total number of article outputs on *Merremia*. The species *M. aegyptia* is a native of South Africa only, which may explain why South Africa is the only African country listed under the top 20 most productive countries with respect to *Merremia* research.

### 3.3. Keyword Analysis

**Figure 1:**
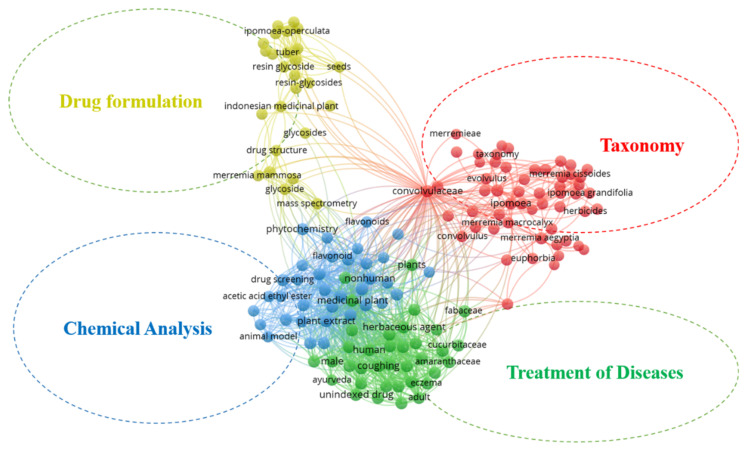
Top keyword co-occurrence networks based on research articles on *Merremia*. Colored clusters represent different research fields, while terms enclosed in colored squares represent the most frequently used keywords.

Keywords reflect the core content of an article, and its analysis in this study aims to recognize the evolving research topic. Figure 1 shows the keyword network generated in the VOS viewer. The keyword network map generated shows the major thematic areas in the literature. Colored clusters represent thematic domains, while words enclosed in large dotted nodes represent the most frequently used terms. Lines between terms show the frequency of occurrence in articles. The map reveals that studies related to *Merremia* species can be grouped into four thematic areas—Drug formulation, taxonomy, chemical analysis, and treatment of diseases. 

### 3.4. Genus Merremia

#### 3.4.1. Taxonomy, Botanical Description, and Distribution

The genus *Merremia* has been reported to be a polyphyletic genus with strong evidence from different molecular analyses [11,30,31]. The taxonomic classification of the genus is provided in Table 3 Approximately 182 species, including infraspecific names, have been reported to belong to the genus *Merremia*, although only 71 correspond to an accepted name, of which 38 are synonyms and 73 listed as unresolved on the Plant List database [32].

*Merremia* species are mostly shrubs or herbs (i.e., herbaceous forbs) with a generally twining or prostrate, but rarely erect growth form. The leaves are entire, lobed, or compound with 3–7 leaflets. Flowers are axillary and solitary or in few- to many-flowered cymes. Bracts are small, ovate or elliptic, and linear. Sepals five, usually subequal, elliptical ovate or ovate-oblong. Corollas are campanulate or funnel-shaped, lobed or entire, yellow or white, sometimes with a dark brown or purple center. Stamens five, filaments filiform, subequal or equal, and are usually widened and glabrous at the base. The anthers are usually twisted with full dehiscence. Pollen grains are smooth and can be 3, 5–6, 9–12-colpate, or 12-rugate. Ovary 2–4-celled, usually with 4 ovules; style filiform; stigmas 2, which are globose or biglobose. Fruits are capsules, usually four-valved that dehisce longitudinally. The seeds are usually 4–6 in number, pubescent, or glabrous. 

**Figure 2:**
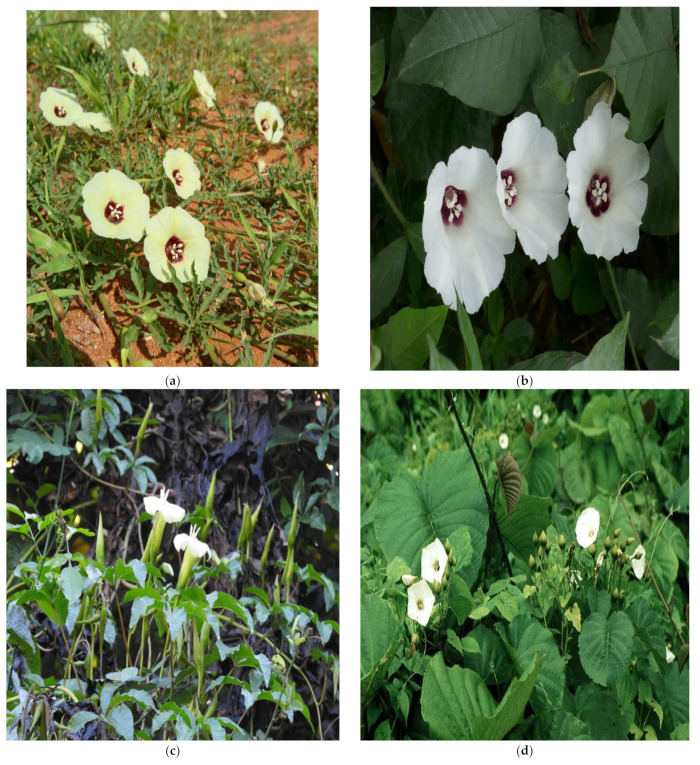

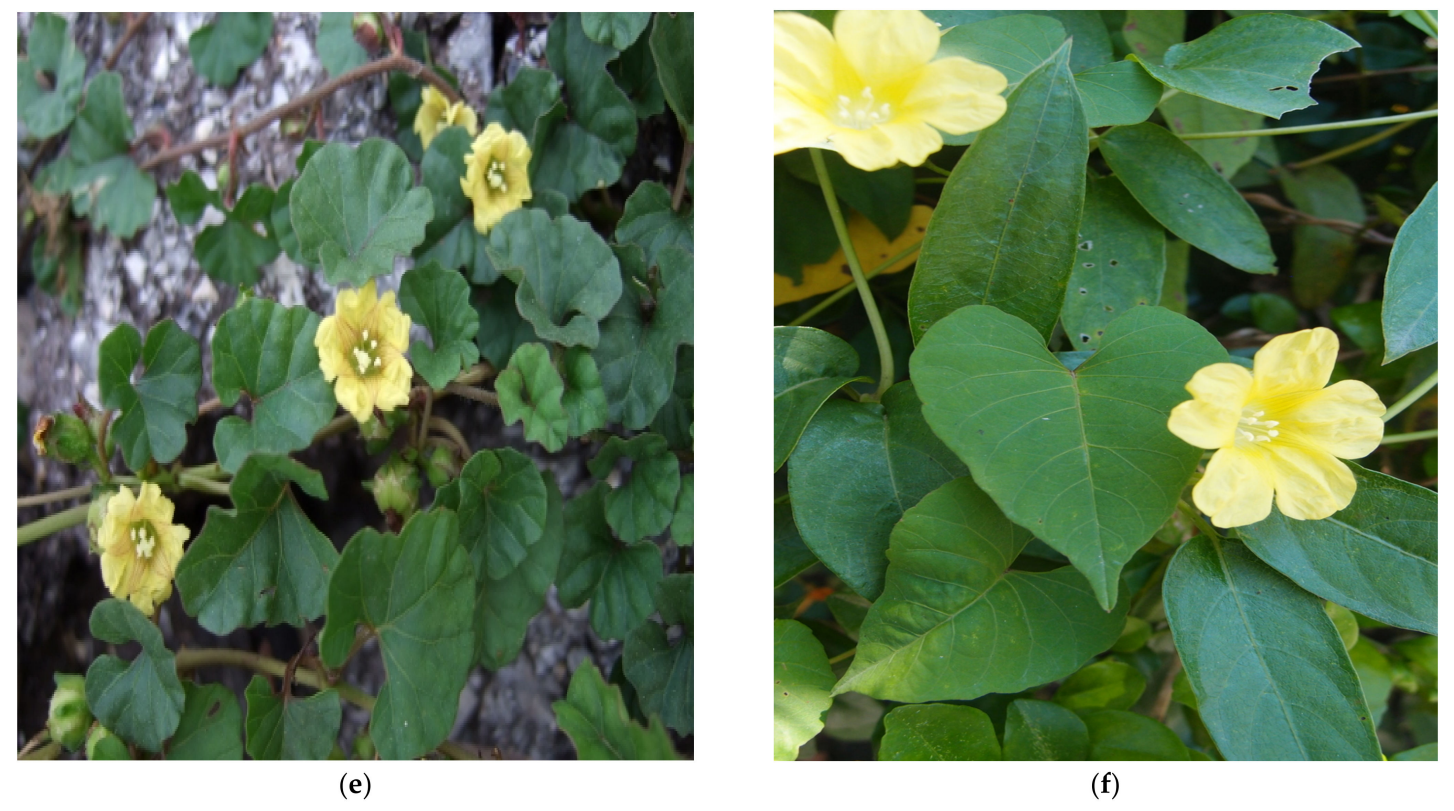
Images of some *Merremia* species. (**a**) *Merremia palmata* Image: © Riana Fourie, (CC-BY-NC) [34]. (**b**) *Merremia pterygocaulos* Image: © Thierrycordenos, (CC-BY-NC) [35]. (**c**) *Merremia platylphylla* Image: © Alexis López Hernández [36]. (**d**) *Merremia peltata* Image: © Matthew Cock [37]. (**e**) *Merremia emarginata* Image: © Convolvulaceae unlimited [38]. (**f**) *Merremia gemella* Image: © Wan-hsuan [39].

Conventionally, species in the genus are recognized by white or yellow flowers with two globos stigmas, anthers that are twisted when flowers are fully opened and functional, and non-spiny pollen grains that could be zonocolpate, tricolpate, pantoporate, pantocolpate, non-spinulose, and valvate fruit dehiscence [10,33] (Figure 2).

**Figure 3:**
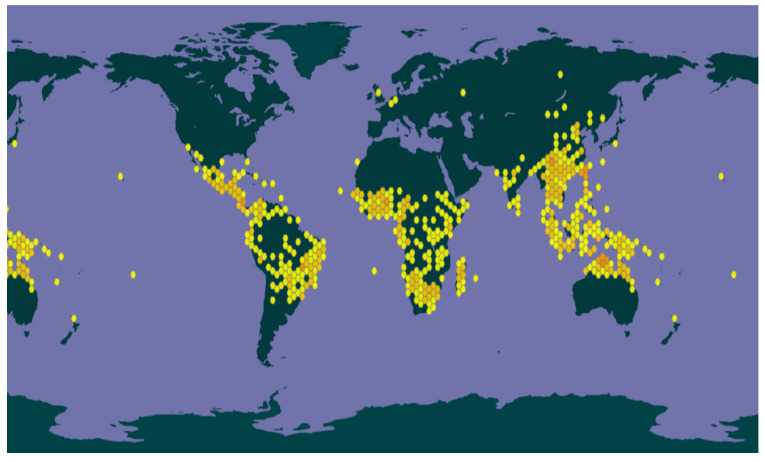
Global distribution of *Merremia* species (Image © OpenStreetMap contributors, GBIF, © OpenMapTiles) [40].

*Merremia* species are distributed in tropical and subtropical regions around the world. *Merremia* is native to Africa, Asia, Australia, North America, and South America. Its full distribution listing is shown in Figure 3 and Supplementary Table S1.

#### 3.4.2. Nutritional Value of *Merremia* Species

Some *Merremia* species are a good source of food and are particularly useful as important fodders for livestock feed. The whole plant of *M. emarginata* is famous for salad in Central Myanmar, Korea [41]. In Argentina, the roots of *M. dissecta* var. *edentata* are used for food by some indigenous people [42]. The leaves of *M. tridentata* are cooked as vegetables in Guinea-Bissau, West Africa [43]. The nutritional analysis of the whole plant of *M. emarginata* revealed important nutritional constituents, which included carbohydrate (63.10%) fat (1.05%), fiber (15.55%), protein (3.28%), ash (7.29%), and moisture (9.73%) [41]. Nunes et al. [44] carried out an ethnobotanical survey of plants used as animal forage in two rural communities in north-eastern Brazil and determined their nutritional and anti-nutritional constituents. *Merremia aegyptia* was listed among the forage plants with a local preference for feeding ruminants given its promotive effect in terms of weight gain and milk production. The analysis of the nutritional composition revealed that *M. aegyptia* has good potential for use in ruminant diets in terms of crude protein contents (20.33 ± 6.26%) and mineral matter (14.06%). The anti-nutritional analysis further suggested that *M. aegyptia* contained condensed tannin and lignin at low concentrations, making it more suitable for inclusion in ruminant diets. 

In another study, *M. tridentata* (synonym: *Xenostegia tridentata*) was included in a feeding trial to investigate its use as a supplementary feed to a common foraged grass, *Panicum maximum* in West African dwarf sheep during the rainy season because of its high acceptability by the animals [45]. In addition, the crude nutrient and tannin contents of *M. tridentata* were analyzed. There was a significant increase in the total food intake in sheep whose ration was supplemented with *M. tridentata* compared with those fed with *P. maximum* only. The crude nutrient analysis revealed higher protein content (15.3% DM) in *M. tridentata* when compared with *P. maximum* (8.6% DM). The tannin content (0.7% DM) of *M. tridentata* did not reduce its palatability and feeding value [45]. 

Galat-Luong et al. [46] studied the diet preferences of the Western giant eland group from the native Sudanian habitat to a wildlife reserve in a Sahelian area. *Merremia pentaphylla* (35.41% feeding bouts) was the second most preferred food item in the list of herbaceous species consumed by the animal. 

In order to develop a suitable feeding strategy for improving grazing sheep production in India, Rajendran and Balakrishnan [47] evaluated the herbage composition, biomass, preference index, and mineral contents in mountain land, fallow land, and waste/roadside land during the Southwest monsoon season. *Merremia tridentata* and *M. emarginata* were listed among the herbage species in the mountain land, fallow land, and waste/roadside land. *Merremia tridentata* had the highest preference index (2.57 ± 2.42) by the sheep in the waste/roadside land. The maximum preference index recorded in *M. tridentata* suggests that the species is more edible than other herbage species consumed by the sheep. In addition, the mineral contents analysis revealed that *M. tridentata* and *M. emarginata*, together with other herbage species contained several minerals such as Ca, Fe, Cu, Zn, Mn, and Co above the critical level, but the phosphorous level was below the critical level.

Collectively from these data, it is evident that *Merremia* species are valuable dietary constituents for humans and particularly for livestock feed. With the ever-increasing human population, changes in consumption patterns caused by raise in income and urbanization, as well as the economic growth of many Asian countries, it is likely that the demand for livestock products will double in the next two decades [48,49]. A major challenge in livestock production in many developing countries is the shortage and fluctuating quantity and quality of feed supply all year round. In addition, livestock is majorly fed on roughages that are of low quality such as sugar cane by-products, cereal straws, all of which contain a high amount of ligno-cellulose materials and are deficient in nutrients (protein, minerals, vitamins, and energy) [49]. These challenges call for supplementation of livestock feed that can improve the low-quality roughages, and contribute to increased profitability and productivity of livestock. Many forb species, including those of the genus *Merremia,* provide high-quality nutrient resources, particularly when there is inadequate forage in the perennial grass-based pasture system [6]. The utilization of *Merremia* species in supplementing livestock feed is therefore encouraged. 

#### 3.4.3. Ethnomedicinal Uses of *Merremia* Species

Various species in the genus *Merremia* have been reported in different parts of the world for the treatment of different diseases. The species *M. tridentata* (20%) and *M. emarginata* (16%) are the most commonly reported, and India has the highest number of research articles reporting the ethnomedicinal uses of *Merremia* species. This may be due to India’s long history of well-developed traditional medical systems (Ayurveda) [50]. 

In India, a decoction made from leaves of *M. emarginata* has been employed in the treatment of fever, neuralgia, urinary infection, rheumatism, liver and kidney diseases [51], inflammation, cough, and headache [21]. The roots of *M. tridentata* prepared by maceration are beneficial in the treatment of diabetes [52] rheumatism, hemiplegia, piles, swellings, and urinary infections [53]. In addition, the whole plant and aerial parts of *M. tridentata*, prepared by maceration have been recorded to be useful in the treatment of leprosy, piles, swellings, rheumatism, stiffness of the joints, hemiplegia, urinary infections [54], and toothache [55].

In Indonesia, the leaf and whole plant of *M. mammosa,* prepared by infusion and maceration, respectively, have been reported to be beneficial in the treatment and management of diabetes [56] and diabetic ulcers [57]. The leaves of *M. peltata* prepared by maceration have been employed in the treatment of different forms of cancer, diarrhea, abdominal pain, cough, sore eyes, wounds, and inflammation [58].

The whole plant of *M. peltata* prepared by maceration has been used as an anti-inflammatory, analgesic, anticancer, anti-viral, anti-malarial, anti-bacterial, and anti-fungal in the Philippines [19].

In Malaysia, the leaves [22] and aerial parts [59] of *M. borneensis* prepared by maceration have been employed in the treatment and relief of breast cancer.

In China, a decoction prepared from the whole plant of *M*. *yunnanensis* has been used to treat typhoid and stroke [60]. An infusion from the leaf and fruit of *M*. *yunnanensis* is taken to treat stroke, hemiplegia, typhoid fever, and headache [61].

In Columbia, the leaves of *M. umbellata* prepared by maceration are used as an antibacterial, antifungal, and anti-inflammatory agent [62].

The leaves of *M. vitifolia* prepared by maceration are used in the treatment of fever, headache, eye inflammation, rheumatism, dysentery, jaundice, and urinary diseases in Bangladesh [20]. The ethnomedicinal uses of the different species in the genus *Merremia* are summarized in Table 4.

Generally, from the reported ethnomedicinal uses of the plant, the herbal formulations of the species are prepared by decoction, maceration, and infusion (Table 4). Furthermore, the leaves of the different species are the most widely utilized (50%) part in ethnomedicine, followed by the whole plant (25.0%), aerial parts (12.50%), roots (8.33%), and fruits (4.16%).

#### 3.4.4. Phytochemistry of Species in the Genus *Merremia*

A wide array of phytochemicals has been identified and isolated from different extracts and fractions of *Merremia* species. Phytochemical investigations have been conducted on the whole plant, the aerial parts, and other individual plant parts including roots and leaves. Most of the reported phytochemicals were found in the roots (Table 5a). However, there are overlapping compounds obtained from different parts of the plant and across species from the genus. Extraction methods are mainly maceration and infusion. The major class of the phytoconstituents widely isolated and identified from this genus is resin glycosides. Other classes of phytoconstituents from this genus are flavonoids, tropane alkaloids, phenolic compounds, isoflavones, coumarins, and sesquiterpenoid. Many of the compounds isolated in the genus *Merremia* are therapeutically relevant, with some used as pharmaceutical ingredients in a few commercial drugs. 

Devadasu et al. [67] carried out phytochemical screening of *M. emarginata* leaves. The results revealed the presence of alkaloids, steroids, glycosides, flavonoids, and phenols. In the same study, a prenylflavonoid, 8-prenylnaringenin (**1**) was isolated from the leaves of *M. emarginata* using silica thin layer chromatography. In addition to the good antioxidant activity displayed by 8-prenylnaringenin (**1**) in the study, this compound is a known phytoestrogen and also has anticarcinogen potential [68].

Phytochemical investigations conducted on the root of *M. yunnanensis* for the first time led to the isolation and structure elucidation of two new eudesmane derivatives viz: 1*α*,6*β*,9*β*-trihydroxy-eudesm-3-ene-1-*O*-*β*-d-glucopyranoside (**29**) and 1*α*,6*β*,9*β*-trihydroxy-eudesm-3-ene-1-(6-cinnamoyl)-*O*-*β*- d-glucopyranoside (**30**) [60]. In another recent study, phytochemical investigations were conducted on *M. yunnanensis* (root and leaves) [61]. This study led to the isolation of a new compound, namely 1*α*,4*β*,8*β*,9*β*-eudesmane-tetrol-1-*O*-*β*-d-glucopyranoside (**31**), together with the isolation of other known compounds viz: tyrosol (**35**), hydroxypinoresinol (**58**), scopoletin (**42**), hydroxycoumarin (**43**), quercetin-7-*O*-glucoside (**3**), and 2-*C*-methylerythritol (**6**). The researchers further reported that compounds (**6**), (**43**), and (**58**) were reported for the first time in the genus *Merremia*, and compounds (**31**) and (**35**) were isolated and reported for the first time in the family Convolvulaceae. The results imply that the compounds isolated in *M*. *yunnanensis* can be considered important chemotaxonomic markers for *M*. *yunnanensis.* Some of the isolated compounds in *M. yunnanensis* are therapeutically relevant. For example, tyrosol (**35**) is well known as a strong antioxidant [69]. Antioxidants have health benefits, quenching reactive oxidants [70,71] and slowing down cell damage/death and inflammation. Quercetin-7-*O*-glucoside (**3**) has been reported to suppress pancreatic cancer by inhibiting the epidermal growth factor receptor (EGFR) signaling in the cell [72]. Rutin has been reported to inhibit SARS-CoV-2 main protease proteins and could be a potential cure for SARS-CoV-2 [73].

In another study, five new pentasaccharide resin glycosides, named merremins A−E (**7**–**10**), (**13**), two new pentasaccharide resin glycoside methyl esters, named merremins F (**16**) and G (**17**), and four known resin glycosides, murucoidin IV (**12**), murucoidin V (**14**), stoloniferin IV (**11**), and murucoidin XVII (**15**) were isolated for the first time from the aerial parts of *M. hederacea*. All these compounds showed multi-drug resistance reversal activities when evaluated further [74]. 

**Figure 4:**
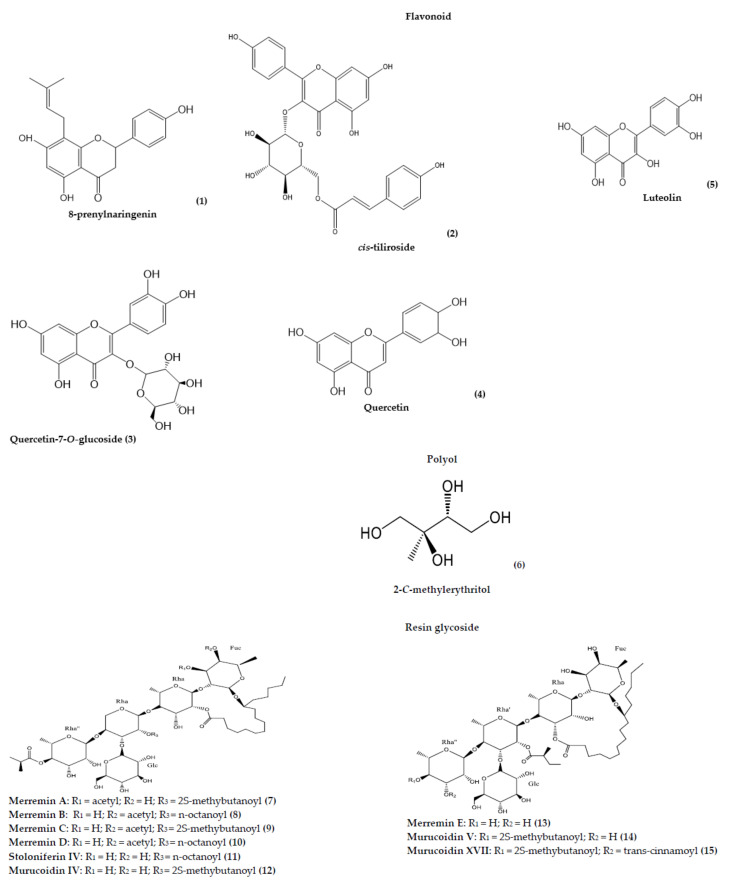

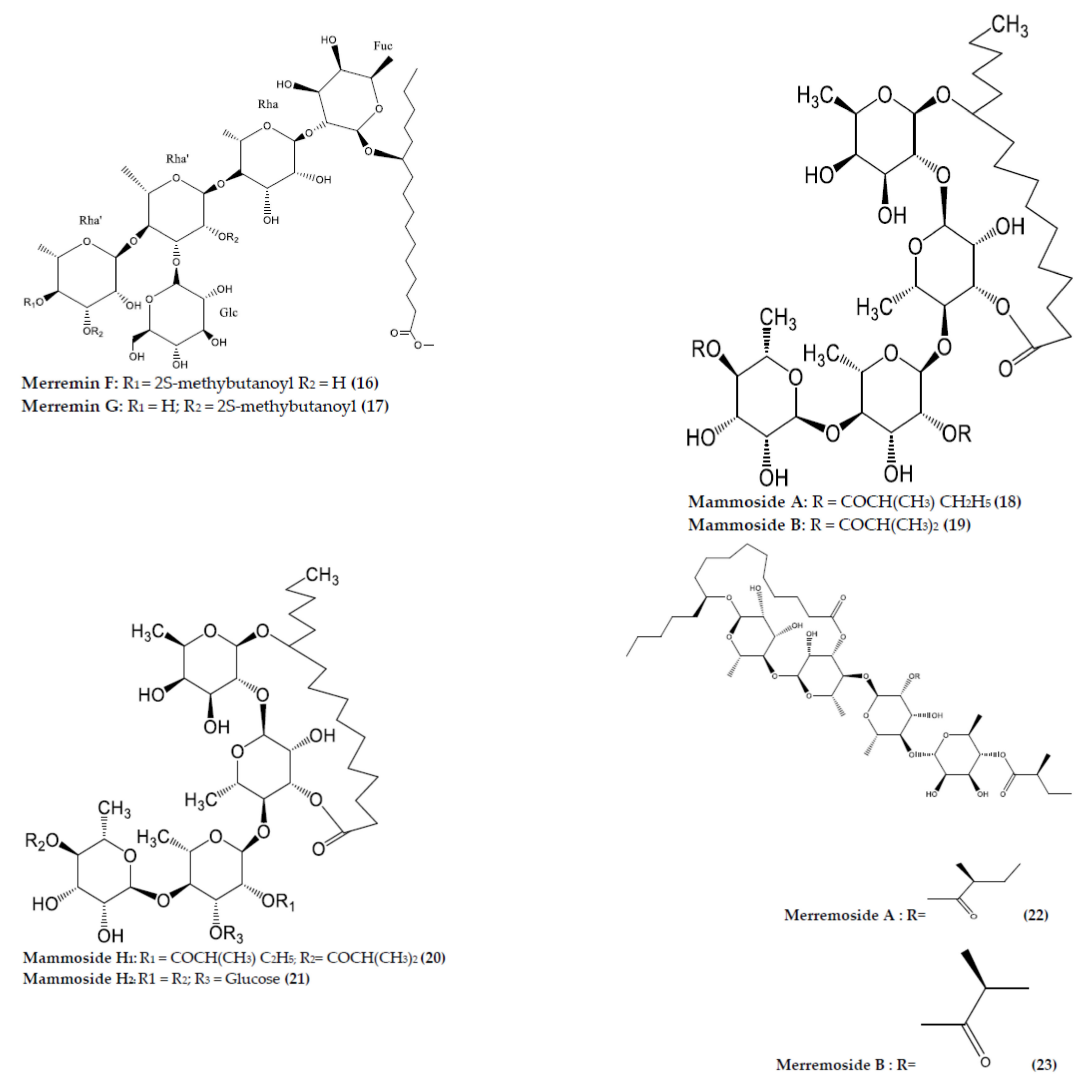

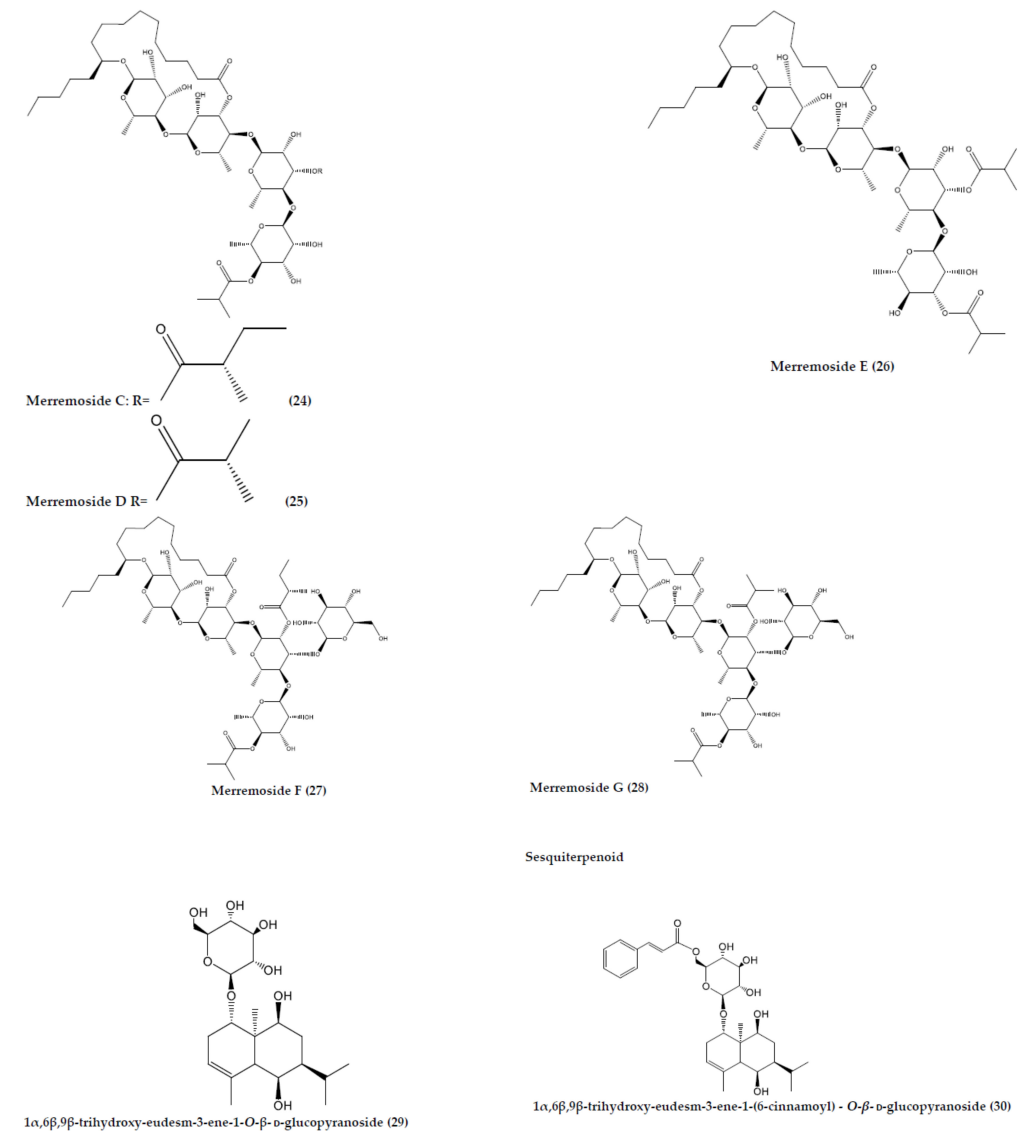

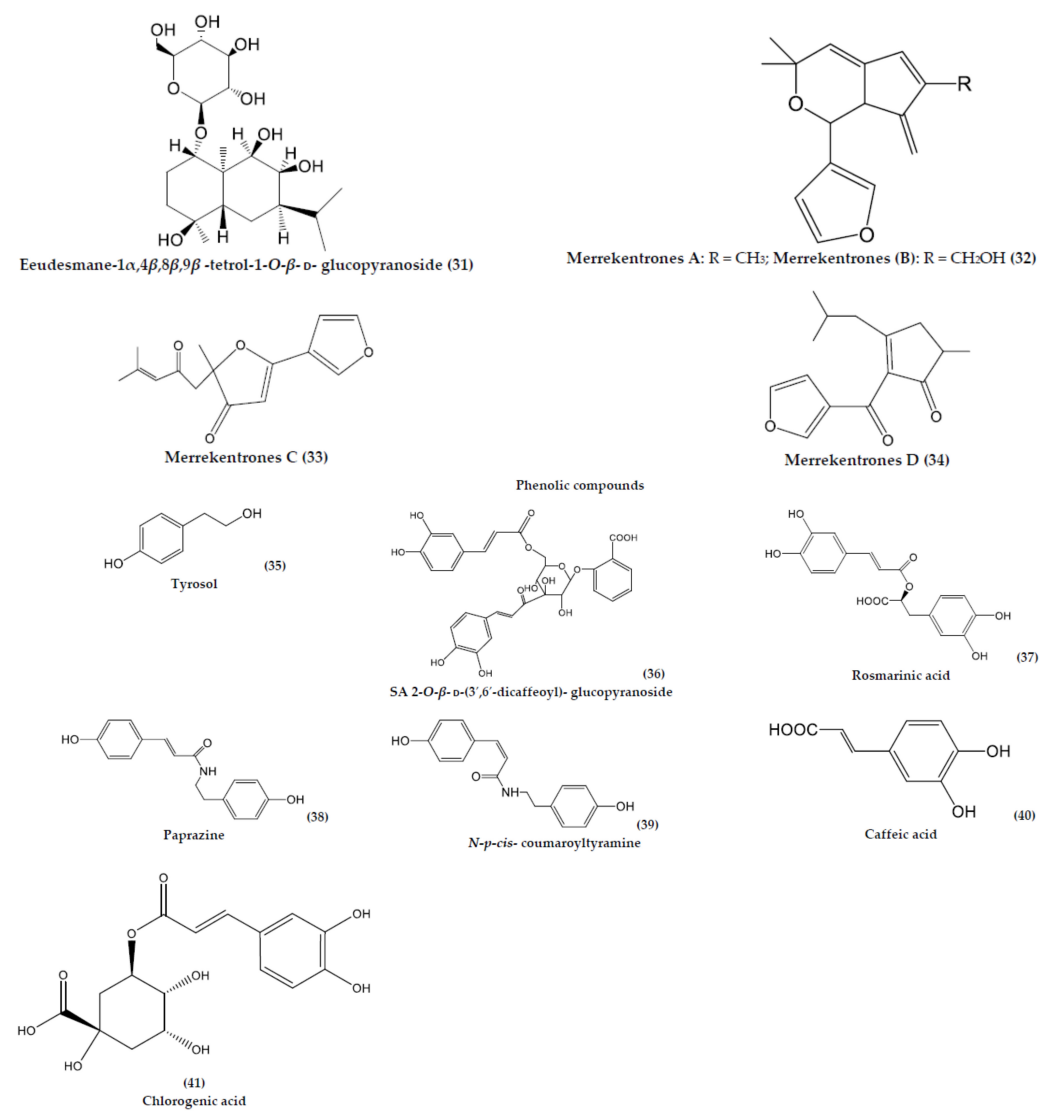

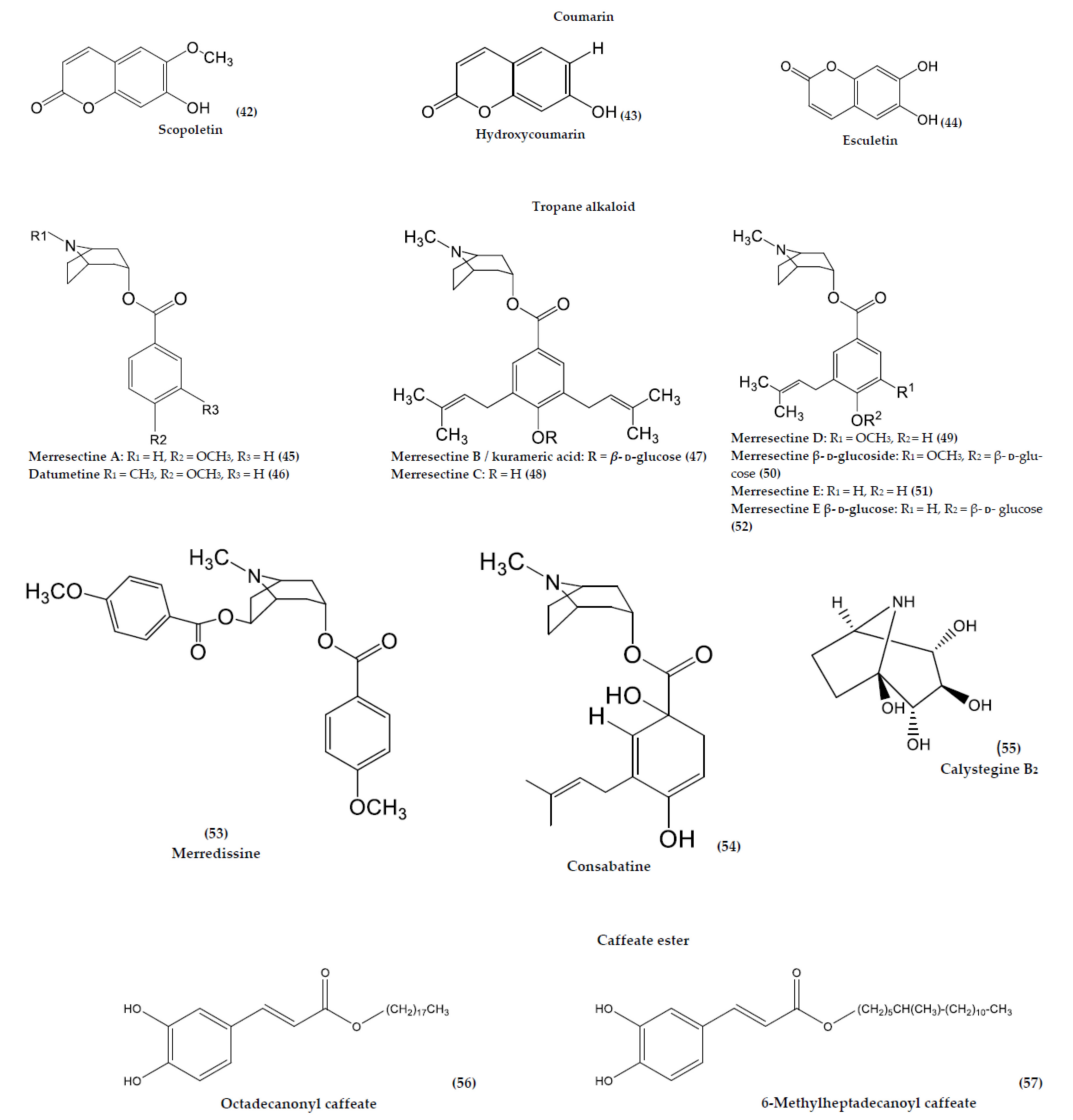

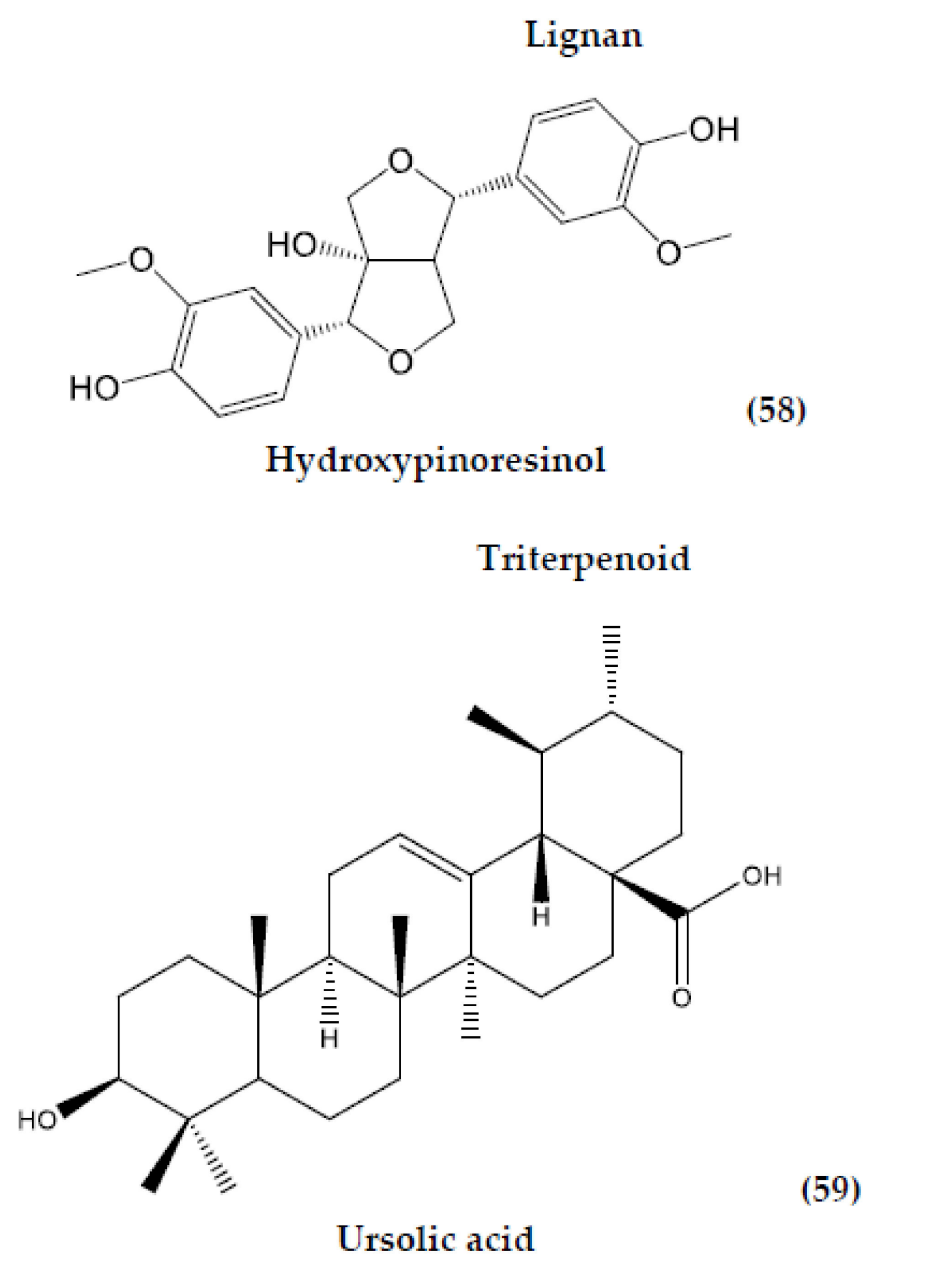
Structures of isolated phytochemicals in the genus *Merremia*.

A chemotaxonomy study of the genus *Merremia* (using 18 species) based on the distribution of tropane alkaloids revealed a total of 74 tropanes and 13 pyrrolidines based on GC-MS analysis [15]. This study further led to the isolation and structure elucidation of four new aromatic 3α-acyloxytropanes named merresectines A–D (**45**,**47**–**49**), (from the roots of *M. dissecta* and *M. guerichii*), one new 3α,6*β*-di-(4-methoxybenzoyloxy) tropane (named merredissine (**53**)) from *M. dissecta*, and datumetine (**46**) (from *M. dissecta* and *M. guerichii*). The results from this study led to the grouping of *Merremia* species into three chemo-taxonomical categories: (1) taxa free of tropanes, (2) taxa with simple tropanes, and (3) taxa with merresectines in addition to simple tropanes. This grouping contributes to the solution of infrageneric taxonomic problems in the genus. According to the study of Rahman et al. [73], caffeic acid derivatives (CAFDs) isolated in *M. umbellata* [64,75] were reported to inhibit SARS-CoV-2 [76]. Esculetin (**44**) and luteolin (**5**) isolated in *M. umbellata* [75] were reported to decrease angiotensin-converting enzyme 2 (ACE-2) expression, and could therefore reduce SARS-CoV-2 infection [77]. However, rosmarinic acid (**37**) isolated in *M. umbellata* [64,75] was reported to increase the expression of ACE-2, which could aggravate SARS-CoV-2 infection [77]. Shimming et al. [14] reported the presence of calystegine B_2_ (**55**) in the leaves and flowers of *M. dissecta*. This compound has been reported to have antidiabetic properties as it was a potent inhibitor of *R*-galactosidases and α-glucosidases [78]. Using ultra-performance liquid chromatography-tandem mass spectrometry (UPLC-MS/MS) and reverse phase-high performance liquid chromatography (RP-HPLC), Angappan et al. [79] identified chlorogenic acid (**41**) in the aqueous leaf extract of *M. emarginata*, which was attributed to the diuretic activity of the extract. In another study, Santos et al. [80] isolated ursolic acid (**59**) and *cis*-tiliroside (**2**) from *M. tomentosa* leaf extract, and these compounds were thought to be responsible for the insecticidal activity of the extract. The medicinal characteristic of the species of *Merremia* and other medicinal plants depends on the structure, quantity, and quality of the phytochemical constituents. The phytochemicals detected and isolated in the genus *Merremia*, alongside their structures are summarized in Table 5, and Figure 4.

**Table 5:** Phytochemicals isolated from the genus *Merremia*.

Species	Isolated Compounds	Molecular Formula	Class of Isolated Compound	Part	Extraction Type	Bioactivity of the Tested Isolated Compound	Reference
*M. emarginata*	8-prenylnaringenin (**1**)	C_20_H_20_O_5_	Flavonoid	Leaf	Maceration	Antioxidant and antimycobacterial activities	[67]
	Chlorogenic acid (**41**)	C_16_H_18_O_9_	Phenolic compound	Leaf	Maceration	Diuretic activity	[79]
*M. hederacea*	Merremin A (**7**)	C_58_H_98_O_26_	Resin glycoside	Aerial	Infusion	Multidrug resistance reversal activity	[74]
	Merremin B (**8**)	C_61_H_104_O_26_	Resin glycoside	Aerial	Infusion	Multidrug resistance reversal activity	[74]
	Merremin C (**9**)	C_59_H_98_O_26_	Resin glycoside	Aerial	Infusion	Multidrug resistance reversal activity	[74]
	Merremin D (**10**)	C_63_H_108_O_26_	Resin glycoside	Aerial	Infusion	Multidrug resistance reversal activity	[74]
	Murucoidin IV (**12**)	C_57_H_98_O_29_	Resin glycoside	Aerial	Infusion	Multidrug resistance reversal activity	[74]
	Stoloniferin IV (**11**)	C_61_H_106_O_25_	Resin glycoside	Aerial	Infusion	Multidrug resistance reversal activity	[74]
	Merremin E (**13**)	C_51_H_88_O_24_	Resin glycoside	Aerial	Infusion	Multidrug resistance reversal activity	[74]
	Murucoidin V (**14**)	C_56_H_96_O_25_	Resin glycoside	Aerial	Infusion	Multidrug resistance reversal activity	[74]
	Murucoidin XVII (**15**)	C_65_H_101_O_26_	Resin glycoside	Aerial	Infusion	Multidrug resistance reversal activity	[74]
	Merremin F (**16**)	C_52_H_92_O_25_	Resin glycoside	Aerial	Infusion	Multidrug resistance reversal activity	[74]
	Merremin G (**17**)	C_52_H_92_O_25_	Resin glycoside	Aerial	Infusion	Multidrug resistance reversal activity	[74]
*M. tomentosa*	Ursolic acid (**59**)	C_30_H_48_O_3_	Triterpenoid	Leaf	Maceration	Insecticidal activity	[80]
	*cis*-Tiliroside (**2**)	C_30_H_26_O_13_	Flavonoid	Leaf	Maceration	Insecticidal activity	[80]
*M. yunnanensis*	1α,6β,9β-trihydroxy-eudesm-3-ene-1*-O*-*β*-d-glucopyranoside (**29**)	C_21_H_36_O_8_	Sesquiterpenoid	Root	Decoction	N/A	[60]
	1α,6β,9β-trihydroxy-eudesm-3-ene-1-(6-cinnamoyl)-*O*-*β*-d-glucopyranoside (**30**)	C_30_H_42_O_9_	Sesquiterpenoid	Root	Decoction	N/A	[60]
	Eeudesmane-1α,4*β*,8*β*,9*β* -tetrol-1-*O*-*β*- d -glucopyranoside (**31**)	C_21_H_38_O_9_	Sesquiterpenoid	Root	Infusion	N/A	[61]
	Tyrosol (**35**)	C_8_H_10_O_2_	Phenolic compound	Root	Infusion	N/A	[61]
	Hydroxypinoresinol (**58**)	C_20_H_22_O_7_	Lignan	Leaf	Infusion	N/A	[61]
	Scopoletin (**42**)	C_10_H_8_O_4_	Coumarin	Leaf	Infusion	N/A	[61]
	Hydroxycoumarin (**43**)	C_9_H_6_O_3_	Coumarin	Leaf	Infusion	N/A	[61]
	Quercetin-7-*O*-glucoside (**3**)	C_21_H_20_O_12_	Flavonoid	Root	Infusion	N/A	[61]
	2-*C*-methylerythritol (**6**)	C_5_H_12_O_4_	Polyol	Leaf	infusion	N/A	[61]
*M. umbellata*	SA 2-*O-β*- d -(3′,6′-dicaffeoyl)-glucopyranoside (**36**)	C_31_H_28_O_14_	Phenolic Compound	Whole plant	Maceration	Allelopathic effect on *Arabidopsis* seed germination	[75]
	Rosmarinic acid (**37**)	C_18_H_16_O_8_	Phenolic Compound	Whole plant	Maceration	Allelopathic effect on *Arabidopsis* seed germination	[64,75]
	Paprazine (**38**)	C_17_H_17_NO_3_	Phenolic Compound	Whole plant	Maceration	Allelopathic effect on *Arabidopsis* seed germination	[75]
	*N*-*p*-*cis*-coumaroyltyramine (**39**)	C_17_H_17_NO	Phenolic Compound	Whole plant	Maceration	Allelopathic effect on *Arabidopsis* seed germination	[75]
	Caffeic acid (**40**)	C_9_H_8_O_4_	Phenolic Compound	Whole plant	Maceration	Allelopathic effect on *Arabidopsis* seed germination	[64,75]
	Esculetin (**44**)	C_9_H_6_O_4_	Coumarin	Whole plant	Maceration	Allelopathic effect on *Arabidopsis* seed germination	[75]
	Quercetin (**4**)	C_15_H_10_O_7_	Flavonoid	Whole plant	Maceration	Allelopathic effect on *Arabidopsis* seed germination	[64,75]
	Luteolin (**5**)	C_15_H_10_O_6_	Flavonoid	Whole plant	Maceration	Allelopathic effect on *Arabidopsis* seed germination	[75]
*M. kentrocaulos*	Merrekentrones A and B (**32**)	C_15_H_14_O_3_	Sesquiterpenoid	Root	Maceration	N/A	[81]
	Merrekentrones C (**33**)	C_15_H_16_O_4_	Sesquiterpenoid	Root	Maceration	N/A	[81]
	Merrekentrones D (**34**)	C_15_H_18_O_3_	Sesquiterpenoid	Root	Maceration	N/A	[81]
*M. mammosa*	Mammoside A (**22**) and B (**23**)	C_48_H_82_O_20_	Resin glycosides	Root	Maceration	N/A	[82]
	Mammoside H1 (**20**)	C_54_H_92_O_25_	Resin glycosides	Root	Maceration	N/A	[82]
	Mammoside H2 (**21**)	C_54_H_92_O_25_	Resin glycosides	Root	Maceration	N/A	[82]
*M. quinquefolia*	Merresectine A (**45**)	C_15_H_19_NO_3_	Tropane alkaloid	Root	Maceration	N/A	[15]
*M. cissoides* and M. *quinquefolia*	Merresectine B/kurameric acid (**47**)	C_31_H_45_NO_8_	Tropane alkaloid	Root	Maceration	N/A	[15]
*M. cissoides* and *M*. *quinquefolia*	Merresectine C (**48**)	C_25_H_35_NO_3_	Tropane alkaloid	Root	Maceration	N/A	[15]
*M. cissoides* and *M*. *quinquefolia*	Merredissine (**53**)	C_24_H_27_NO_6_	Tropane alkaloid	Root	Maceration	N/A	[15]
*M. dissecta*	Datumetine (**46**)	C_16_H_21_NO_3_	Tropane alkaloid	Root	Maceration	NA	[15]
*M. guerichii*	Merresectine D (**49**)	C_27_H_39_NO_9_	Tropane alkaloid	Root	Maceration	N/A	[15]
	Merresectine *β*-d–glucoside (**50**)	C_27_H_39_NO_9_	Tropane alkaloid	Root	Maceration	N/A	[15]
*M. quinata*	Consabatine (**54**)	C_20_H_31_NO_4_	Tropane alkaloid	Root	Maceration	N/A	[15]
*M. cissoides* and *M*. *quinquefolia*	Merresectine E (**51**) and Merresectine E *β*-d-glucoside (**52**)	C_20_H_27_NO_3_	Tropane alkaloid	Root	Maceration	N/A	[15]
*M. tuberosa*	Octadecanonyl caffeate (**56**)	C_27_H_44_O_4_	Caffeate ester	Root	Maceration	NA	[83]
	6-Methylheptadecanoyl caffeate (**57**)	C_27_H_42_O_5_	Caffeate ester	Root	Maceration	NA	[83]
*M. dissecta*	Octadecanonyl caffeate (**56**)	C_27_H_44_O_4_	Caffeate ester	Root	Maceration	NA	[83]
	6-Methylheptadecanoyl Caffeate (**57**)	C_27_H_42_O_5_	Caffeate ester	Root	Maceration	NA	[83]
	Calystegine B_2_ (**55**)	C_7_H_13_NO_4_	Tropane alkaloid	Leaf, Flower	N/A	Antidiabetic activity	[14,78]
*M. mammosa*	Merremoside A (**22**)	C_50_H_86_O_20_	Resin glycosides	Tuber	Maceration	NA	[84]
	Merremoside B (**23**)	C_48_H_82_O_20_	Resin glycosides	Tuber	Maceration	NA	[84]
	Merremoside C (**24**)	C_49_H_84_O_20_	Resin glycosides	Tuber	Maceration	NA	[84]
	Merremoside D (**25**)	C_48_H_82_O_20_	Resin glycosides	Tuber	Maceration	NA	[84]
	Merremoside E (**26**)	C_48_H_82_O_20_	Resin glycosides	Tuber	Maceration	NA	[84]
	Merremoside F (**27**)	C_55_H_94_O_25_	Resin glycosides	Tuber	Maceration	NA	[85]
	Merremoside G (**28**)	C_54_H_92_O_25_	Resin glycosides	Tuber	Maceration	NA	[85]

N/A: Not available.

#### 3.4.5. Biological and Pharmacological Activities

Due to the different ethnomedicinal usage and phytochemicals present in the genus *Merremia*, several biological and pharmacological have been assessed in 11 species (viz. *M. aegyptia, M. borneensis*, *M. dissecta, M. emarginata*, *M. hederacea*, *M. mammosa*, *M. peltata, M. tridentata*, *M. tomentosa*, *M. umbellata*, and *M. vitifolia*). Antimicrobial and antioxidant activities are the most studied, with *M. emarginata* being the most researched species. The various scientific studies documenting the relevant biological activity of *Merremia* species are presented below and summarized in Table 6. The activities are presented in terms of the plant extract types, experimental methodologies, extract concentrations/doses, and possible mechanisms of action where the information was available.

##### Cancer Cell Cytotoxicity

In recent years, research on medicinal plants as potential chemotherapeutic agents has increased globally because of their actions that prevent cancer initiation and proliferation with limited toxicity, as well as their anti-multidrug reversal ability [86,87]. There are very few scientific studies on *Merremia* species as anticancer agents, but their traditional use in treating breast cancer may have prompted investigations on their potential anticancer and antiproliferative properties as discussed below. The antiproliferative activities of three solvent extracts (50 µg mL^−1^ ethyl acetate, hexane, and methanol) from whole plants of *M*. *emarginata* were investigated on human cancer cell lines using the 3-(4,5-Dimethylthiazol-2-yl)-2,5-diphenyltetrazolium bromide (MTT) reduction assay [3]. The results demonstrated antiproliferative effects of the extracts against the cell lines of which the ethyl acetate extract was the most effective, inhibiting the proliferation of cell lines A549, KB, MIA-PaCa-2, and DU-145 with IC_50_ values of 28.5 μg mL^−1^, 37.2 μg mL^−1^, 51 μg mL^−1^, and 69.4 μg mL^−1^, respectively at 50 μg mL^−1^. In another study, the antiproliferative potential of the leaf extracts (methanol, ethyl acetate, and hexane) of *M. emarginata* and different parts of nine other medicinal plants on human cancer cell lines and monkey normal kidney epithelial cells (VERO) was investigated [88]. The leaf extracts were tested at different concentrations (range: 3.13–200 μg mL^−1^) and time intervals (24, 48, and 72 h). The antiproliferative study was done using the MTT assay, while flow cytometry was used to determine apoptosis. The results revealed that *M. emarginata* hexane extract exhibited cytotoxic effects in all the cell lines in a concentration- and time-dependent manner, ranking fifth out of the 10 plants used in the trial. The hexane extract (25 μg mL^−1^) particularly inhibited A549 and COLO 320 DM cell line proliferation (with IC_50_ values of 15.5 μg mL^−1^ and 18.4 μg mL^−1^, respectively) and had minimal toxicity (IC_50_ value of 65.2 μg mL^−1^) for VERO cells at 72 h. The authors speculated that the inhibition of cell line proliferation by *M. emarginata* extract might be due to its antiproliferative potential to demonstrate cancer cell-specific death. Wang et al. [74] tested the cytotoxicity and multidrug resistance reversal activity of different types of isolated pentasaccharide resin glycoside compounds (25 μM of merremins A−G (**7**–**10**, **13**, **16**, **17**) and murucoidin IV (**12**), murucoidin V (**14**), stoloniferin IV (**11**), and murucoidin XVII (**15**)) from *M. hederacea* aerial parts in KB/VCR cell lines using a sulforhodamine B assay. Vinblastine served as a reference. Merremins A (**7**), E (**13**), G (**17**), and murucoidin V (**14**) were non-cytotoxic at 25 Μm but enhanced the cytotoxicity of vinblastine 2.3−142.5-fold at 25 Μm, and demonstrated anti-multidrug reversal activity. Taken together, the results suggest that the investigated extracts and compounds from *Merremia* species have the potential to be developed into chemotherapeutic agents as they showed significant cytotoxic effects and inhibited the proliferation of cancer cells. However, more in vivo studies and clinical trials are required. Other *Merremia* species must be investigated for their anticancer activities, particularly those species (*M. borneensis*, *M. peltata* and *M. mammosa*) that have been reported to be used in treating cancer in traditional medicine.

##### Anti-Diabetic Activity

Diabetes is a chronic disease marked by high blood sugar resulting from a decrease in insulin production by pancreatic beta cells, or when the body cannot effectively use the insulin it produces [89,90]. In 2019, diabetes was the direct cause of 1.5 million deaths globally, and it has been projected that diabetes will be the seventh leading cause of death by 2030 [89]. Additionally, diabetes has been associated with increased mortality and severity of COVID-19 disease [91,92]. Several alpha-glucosidase inhibitor drugs (such as Miglitor Acarbose, and Voglibose) are used in the treatment of diabetes [90]. Several in vivo and in vitro studies have reported that members of the genus *Merremia* demonstrated inhibitor activity of alpha-amylase and alpha-glucosidase enzymes. In vitro studies reported that the ethanolic and ethyl acetate leaf and stem extracts of *M. peltata* (IC50 value 47.44–72.85 μg/mL) [90], the ethanolic leaf extract of *M. hederacea* (91.44% inhibition) [18], and the hexane fraction of *M. mammosa* (66.19 ± 0.41% inhibition) [56] demonstrated good alpha-amylase inhibition. In all these studies, extracts from *Merremia* species had higher *α*-glucosidase inhibition compared to the reference anti-diabetic drug (acarbose). In vivo studies using streptozotocin-induced diabetic male Wistar rats reported that the aqueous extract of *M. tridentata* roots [52], methanolic extract of M. *emarginata* whole plant [93], and ethanolic leaf extract of *M. hederacea* [18] demonstrated potent antidiabetic activities in streptozotocin-induced diabetic rats that were comparable to the reference drug (glibenclamide) used. Overall, the anti-diabetic effects exhibited by extracts of the *Merremia* species studied were attributed to their phytochemical constituents particularly flavonoids and phenolic compounds. Although the results of the in vitro and in vivo studies corroborate the ethnomedicinal claims of *Merremia* species as antidiabetic plants, it is recommended that longer duration studies on the chronic model are carried out to explicate the mechanism of action in order to develop these species as potential antidiabetic drugs. 

##### Antimicrobial Activities

Several studies have shown that *Merremia* species have antibacterial and antifungal effects, and thus have the potential to be developed as antimicrobial agents. Different parts of *Merremia* species extracts show inhibitory actions on several bacteria and fungi to different degrees. The antibacterial activity of the methanolic leaf extracts of Convolvulaceae family members, including *M*. *tridentata* was assessed against two Gram-positive bacteria (*Bacillus subtilis* and *Staphylococcus aureus*) and two Gram-negative bacteria (*Escherichia coli* and *Vibrio parahaemolyticus*) [94]. The extracts had more antibacterial activity on Gram-positive bacteria (MIC range: 0.25–0.5 mg mL^−1^; zone formation range: 10 mm‒14 mm) than Gram-negative bacteria (MIC range: 0.5–1 mg mL^−1^; zone formation range: 9 mm‒11 mm). Pavithra et al. [66] also reported that the methanol stem extracts of *M. tridentata* showed strong antibacterial activity only on Gram-positive bacteria (*S. aureus* and *B. subtilis*) with inhibition zones of 12.0 mm and 11.3 mm, respectively. These extracts were evaluated for antibacterial activity using the broth dilution and disk diffusion assays. The extracts demonstrated antibacterial effects comparable to the standard drugs (ampicillin and gentamicin). The bactericidal effect was attributed to the extract’s phytochemical constituents like alkaloids, triterpenoids, tannins, glycosides, and steroids. In another study, the aqueous, petroleum ether and methanol extracts (concentration range of 10 µg mL^−1^, 25 µg mL^−1^, and 50 µg mL^−1^, 100 µg mL^−1^) of *M. emarginata* leaves showed antibacterial activities against *B. cereus*, *E. coli*, *P. aeruginosa*, and *S. aureus* [95]. The leaf extracts exhibited higher antibacterial activity (with a zone of inhibition range of 0.7–13.7 mm) on all the bacteria compared with penicillin (16.0–17.0 mm). The observed antibacterial activity of *M. emarginata* leaf extracts was ascribed to the presence of chemical compounds such as flavonoids, glycosides, terpenoids, starch, and amino acids in the aqueous extract; flavonoids, tannins, carbohydrates, and amino acids in the methanol extract, and flavonoids, tannins, glycosides, carbohydrates, and amino acids in the petroleum ether extract. Diwan and Gadhikar [96] reported that all four extract solvents (aqueous, acetone, chloroform, and petroleum ether) of *M. emarginata* leaves were effective against bacteria (*A. viscoscus*, *B. subtilis*, *E. coli*, *L. rhamnosus*, *S. aureus*, *S. epidermidis*, and *S. mutans*) known to cause oral diseases in human. Acetone extract had maximum inhibitory (24%) action against *S*. *epidermidis* and was the most effective. The extracts’ antibacterial properties were imputed to their aromatic phytochemical contents (e.g., phenolics).

Using Luria-Bertani agar disk diffusion assay, Rameshkumar et al. [21] investigated the antibacterial activity of *M. emarginata* aqueous leaf extract, thioglycolic acid-capped cadmium telluride quantum dots (TGA-capped-CdTe QDs, hereafter), and their mixture (T-M complex, hereafter) (1 × 10^−6^ M and 100 µg, respectively) against a Gram-negative bacterium, *E. coli*. The T-M complex served as an antimicrobial agent. The results showed that the plant extract alone and T-M complex showed 10 mm and 16 mm zones of inhibition, respectively. Compared with *M. emarginata* extract, the antibacterial activity of the T-M complex was thought to be due to electrostatic interactions. The observed antimicrobial activity of the T-M complex was ascribed to the capability of TGA-capped-CdTe QDs and *M. emarginata* extract phytochemical constituents (e.g., alkaloids, flavonoids, phenolics, saponins, steroids, and terpenoids) to induce oxidative stress on the bacteria (*E*. *coli*) surface biomolecules thereby damaging its membrane. *Merremia umbellata* methanol leaf extract (1000 μg mL^−1^) showed antibacterial activities with percentage inhibition of 32%, 55%, and 67% on *Klebsiella pneumoniae* (13883), *S. aureus* (25923), and *Pseudomonas aeruginosa* (27853), respectively [62]. The observed antibacterial effect was ascribed to phytochemicals such as leucoanthocyanidins, terpenes, and/or steroids and tannins present in the extract. The ethanol leaf extract of *M. peltata* showed a 5.7-mm average zone of inhibition against *B. subtilis* and *S. aureus* (at 10 µg mL^−1^ and 20 µg mL^−1^, respectively), relative to the 2 mm by streptomycin. *M. peltata* ethanol leaf extract had 4.7-mm and 2.7-mm average zones of inhibition against *P. aeruginosa* and *E. coli* (at 15 µg mL^−1^ and 5 µg mL^−1^, respectively), relative to the 1 mm by chloramphenicol [19].

Luciardi et al. [4] reported that *M. dissecta* methanol leaf extracts showed a high antibacterial effect against *P. aeruginosa* and biofilm inhibition effects against *P. aeruginosa* and *S. aureus*, compared with azithromycin which served as the control. The observed antibacterial activity was ascribed to lipophilic compounds present in the extract, suggesting that the extract was toxic to cell membranes by causing loss of chemiosmotic control. It was suggested that volatile plant metabolites, like spathulenol, present in the extracts may be responsible for decreasing the biosynthesis of N-acyl homoserine lactone by 72%, attenuating the expression of virulence factors such as elastase activity (27%) and biofilm production (55%) in *P. aeruginosa*. 

Studies on antifungal activities have only been done on *M*. *tridentata* and *M. borneensis*. In both studies, although the antifungal activities were tested on mould and yeast fungal strains, both species only showed antifungal activity on yeast. The methanol leaf extract of *M*. *tridentata* (at 0.01 mg mL^−1^, 0.05 mg mL^−1^, 0.1 mg mL^−1^, 0.25, 0.5 mg mL^−1^ and 1 mg mL^−1^) showed antifungal activity on yeast (MIC range: 0.25–0.5 mg mL^−1^; zone formation range: 10–15 mm) [94]. Zulhamizan et al. [63] reported that methanol leaf extract of *M. borneensis* showed antifungal activity against *S. cerevisiae* with increasing concentration (60–400 mg mL^−1^) and the inhibition zones ranged between 5.5–15.5 mm. The observed antifungal activity was attributed to the presence of palmitic acid in the methanol leaf extract only.

The above studies suggest that *Merremia* species possess antimicrobial (antibacterial and antifungal) properties. However, the studies are in vitro studies and may not show the same effects in in vivo investigations. For more reliable and valid results, the antimicrobial activities of *Merremia* species extracts should be investigated in in vivo models infected with clinically isolated strains and specific microorganisms to demonstrate efficacy and elucidate their mechanism of action.

##### Anti-Influenza Activity

Influenza is a highly transmissible and widespread respiratory disease that is caused by influenza viruses and occurs throughout the year in tropical areas [97]. According to WHO, global influenza epidemics result in 3–5 million severe illness cases and 290,000-650,000 mortalities yearly [98]. Several anti-influenza viral drugs including Zanamivir are used for treating influenza viral infection. However, the emergence of resistance of viruses to these drugs, as well as adverse side effects, has been reported [97]. Natural products with good tolerability and greater efficacy than existing anti-influenza drugs are of interest in the treatment of influenza virus infection. Only one report is available on the anti-influenza activity of *Merremia* species. Using hemagglutinin assay, the anti-influenza A (subtype H1N1) activity of ethyl acetate fraction of methanol extract of *M. mammosa* tuber was evaluated on embryonic chicken eggs injected with the H1N1 virus [99]. The virus subtype H1N1 (with no treatment extract) and Zanamivir (10 μg mL^−1^) served as negative and positive controls, respectively. The result showed that the ethyl acetate fraction of the *M. mammosa* tuber extract (1000 μg mL^−1^) had an anti-influenza effect similar to Zanamivir, reducing hemagglutinin virus titer by 94% and 100%, respectively. The activity was attributed to the flavonoid and terpenoids contents of the ethyl acetate fraction. The result indicates that *M. mammosa* tuber extract may be a potential alternative medicine for the treatment of the influenza virus. However, this is an in vitro study, in vivo studies are required to evaluate whether *M. mammosa* tuber extract will result in antiviral activity in vivo and to elucidate the mechanism of action. Furthermore, other species in the genus should be investigated for their anti-influenza activities. 

##### Anti-Inflammatory Activity

Inflammation is a defense mechanism of the body’s immune system against unwanted foreign substances, tissue injury, or pathogens that attack tissue cells. However, unregulated inflammation often results in chronic inflammation, consequently leading to the development of various chronic cardiovascular and pulmonary diseases [100]. Several pro-inflammatory mediators such as inducible cyclooxygenase enzyme (COX-2) and tumor necrosis factor-alpha (TNF—α) are usually observed in numerous inflammatory diseases, including inflammatory vascular diseases and rheumatoid arthritis [101]. Medicinal plants that can selectively and effectively inhibit the expression or activation of these pro-inflammatory mediators are of significant clinical importance. The anti-inflammatory activity of orally administered whole plant *M. tridentata* extracts (water, ethanol, benzene, petroleum ether, and chloroform) on male albino rats using the carrageenan-induced rat paw inflammation method was assessed [55]. Normal saline and indomethacin were used as the control and standard drug, respectively. The extracts inhibited paw volume at a range of 15.6–38.3%; the ethanol extract was the most effective with 38.3% and 42.8% inflammation inhibition at 100 mg kg^−1^ and 200 mg kg^−1^ doses, respectively, relative to that the standard drug (48.5%) after 3 h. The ethyl acetate extract of *M*. *emarginata* at a concentration of 50 μg mL^−1^ effectively inhibited TNF-α production (IC_50_ of 5.9 μg mL^−1^) in lipopolysaccharide (LPS)-induced human acute monocytic leukemia cells (THP-1) [3]. In another study, the anti-inflammatory activity of different extracts (250 mg kg^−1^ of petroleum ether and 300 mg kg^−1^ of ethyl acetate, solvent ether, butanol, and butanone) fractionated from *M. tridentata* ethanol roots extract was tested by the authors of [53] in albino mice using the carrageenan-induced rat paw edema method. Orally administered Tween 80 solution and aspirin served as the control and standard drug, respectively. The results showed that solvent ether, ethyl acetate, butanol, and butanone fractions were more effective (87%, 84%, 72%, and 68% at 300 mg kg^−1^, respectively) at inhibiting rat paw edema, relative to the standard (59.7%) and control (no inhibition). The anti-inflammatory activity was attributed to the antimicrobial property of flavonoids present in the extracts. Using the complete Freund’s adjuvant-induced arthritis model, Kamalutheen et al. [55] reported that the methanolic extract of *M*. *tridentata* whole plant demonstrated 49.0% and 51.7% anti-arthritic activity at 100 mg kg^−1^ and 200 mg kg^−1^ on male albino rats. The anti-arthritic activity of the extract was comparable to the standard drug (indomethacin) (55.5%). In another in vitro anti-arthritic study using the protein denaturation inhibition method, at 250 μg mL^−1^, the ethanol extract of *M. emarginata* whole plant and its methanol and ethyl acetate fractions exhibited high protein denaturation inhibition percentages of 95.4%, 87.7%, and 72.6%, respectively, which was comparable with the standard (diclofenac sodium) (98.4%) [102]. However, using this same protein denaturation inhibition technique in a recent study, the aqueous portion of fractionated methanol extract (APFME) of *M. vitifolia* leaves only showed anti-arthritic activity of 64% at a higher concentration of 500 μg mL^−1^ [20].

Collectively, these findings demonstrate that the investigated *Merremia* species extracts possess remarkable in vitro and in vivo anti-inflammatory activities, which support the traditional use of some of these species in the treatment of various inflammatory diseases.

##### Anti-Nociceptive Activity

Pain is an unpleasant sensory and emotional experience linked with or resembling that related to actual or potential tissue damage [103]. To relieve pain, non-steroidal anti-inflammatory drugs such as salicylates and acetic acid derivatives, and opioids are used. However, the prolonged use of these medications leads to decreased gastrointestinal system function [104]. Antinociceptive drugs developed from plant extracts with little or no side effects are of interest in modern medicine. Using formalin-induced paw licking (at early and late stages) and acetic acid-induced writhing tests, the in vivo antinociceptive activity of *M. vitifolia* APFME (200 mg kg^−1^ and 400 mg kg^−1^ BW, PO) was assessed in Swiss Albino mice [20]. Diclofenac sodium (10 mL kg^−1^ BW, administered intraperitoneally (IP)) served as the standard drug. In the formalin-induced paw licking test, the findings of the study demonstrated that the APFME at 200 mg kg^−1^ and 400 mg kg^−1^ BW PO showed dose-dependent higher anti-nociceptive activity (44% and 26%, 30%, and 20% in both early and late test stages, respectively) relative to the control (20% and 15%, respectively) at the same concentrations. Similarly, reduced (44% and 30%, respectively) abdominal contortions with increasing doses of APFME relative to the control (21%) were observed in the acetic acid-induced writhing test. Future studies should investigate more species in the genus for their antinociceptive activity and attempt to identify pure compounds and elucidate the mechanism of action responsible for the activity.

##### Thrombolytic Activity

Several thrombolytic drugs including streptokinase, are used to treat acute coronary disorders resulting from thrombosis [20]. However, there are reports of hypertension and severe hemorrhagic transformation resulting from the use of thrombolytic drugs [20]. Alternative medicines from medicinal plants with thrombolytic activity are increasingly being researched for their safety profile to overcome the side effects resulting from modern thrombolytic drugs. Only one report, however, is available on the thrombolytic activity of *Merremia* species. The aqueous portion of fractionated methanol extract of *M. vitifolia* leaves (500 μg mL^−1^) was reported to demonstrate significant thrombolytic activity (42.5% clot lysis) in blood samples from male and female adult human volunteers as compared with the negative control (normal saline; 4.80%) [20]. However, the positive control (streptokinase) demonstrated much higher thrombolytic activity (72.2%) than *M. vitifolia*. This study was assessed in blood samples from male and female (1:1) adult human volunteers with no anticoagulant and oral contraceptive treatments history. The inhibition of clot formation in the subjects was attributed to various phytochemicals (alkaloids, flavonoids, tannins, and triterpenoids) in *M. vitifolia*. Studies to identify the major compound responsible for this activity, as well as its mechanism of action, should be conducted. Although *M. vitifolia* leaf extract gave low thrombolytic activity as compared with the positive control in this study, other species in the genus may give higher thrombolytic activity if evaluated. Future studies should evaluate these species for thrombolytic activity.

##### Anti-Urolithiatic Activity

Urolithiasis (commonly referred to as kidney stones) is the formation of solid particles anywhere in the urinary tract and it is the most prevalent type of all urinary stone diseases [105,106]. Usually, the solid particles are very small and can dissolve and leave the body without any problem. However, the blockage of the flow of urine by even a small stone results in excruciating pain, which requires immediate medical attention [106]. Several pharmaceutical drugs and medical technologies such as percutaneous nephrolithotomy and extracorporeal shock wave lithotripsy are available to treat and prevent urolithiasis. However, these medications and techniques are expensive and there are risks of side effects and reoccurrence of urolithiasis even with medication [106]. Several medicinal plants, including *Merremia* species, have the potency to inhibit stone formation as well as break formed stones.

A dissolution model study was carried out by Neeraja Kamakshi et al. [107] on the anti-urolithiatic activity of methanol extracts of the *M*. *emarginata* plant. Artificial stones (calcium phosphate and calcium oxalate) were produced by homogenous precipitation, while eggs’ semi-permeable membrane served as dissolution bags; Cystone^®^ served as a positive control. *M*. *emarginata* showed anti-urolithiatic activity in a dose-dependent manner (with calcium phosphate and calcium oxalate mineralization inhibition of 81% and 84%, respectively, relative to 91% by the standard drug at 400 µg mL^−1^). The observed bioactivity was attributed to the presence of phytochemicals such as alkaloids and flavonoids in the plant extract. The result implies that the methanolic extract of the *M. emarginata* plant has great potential to be developed as a pharmaceutical agent for the treatment of urolithiasis. However, in vivo and clinical studies are required to clarify the mechanism of action and identify the exact compound responsible for this activity. Other species in the genus should be investigated for their anti- urolithiatic activities.

##### Nephroprotective Activity

Nephrotoxicity usually results from the frequent use of therapeutic and chemotherapeutic drugs like aminoglycoside antibiotics (gentamicin) and cisplatin, chronic diseases, and exposure to heavy metals. These causes often result in End-Stage Renal Disease (ESRD) and death. The few therapeutic drugs currently available to treat nephrotoxicity only delay and do not stop the progression of nephrotoxicity [108]. There is increasing research focus on medicinal plants with nephroprotective activities that can be used as safer and better alternatives in treating nephrotoxicity. The nephroprotective activity of ethanol extract of *M. emarginata* leaves (150, 200, and 250 mg kg^−1^ BW, PO) in adult albino Wistar rats was investigated by Rameshkumar et al. [64] using a histopathology examination. Nephrotoxicity was induced with gentamicin (20 mg kg^−1^ BW, IP); normal drinking water (10 mg kg^−1^ BW) served as control. The results revealed that the extracts caused regeneration of glomerular, tubular, and proximal tubular epithelial cells of damaged kidneys in *M. emarginata* extract-treated rats (especially at 250 mg kg^−1^). The leaf extract’s nephroprotective activity was ascribed to its antioxidant (polyphenolics) contents. The result supports the traditional use of *M*. *emarginata* leaves in treating kidney disorders. Further studies should be carried out to determine the exact polyphenolics and their mechanism against nephrotoxicity. Other *Merremia* species (*M. tridentata* and *M. vitifolia*) that are used in traditional medicines to treat various kidney and urinary disorders should be investigated for their nephroprotective activities. 

##### Diuretic/Blood Pressure-Lowering Activity

Diuretics are medications that elevate the amount of water and salt expelled from the body as urine and are widely used in the treatment of kidney and liver diseases, edema, hypertension, and congenital heart failure [79]. Commercial diuretic drugs have been reported to be associated with many side effects (such as dehydration, hypokalemia, fever, bleeding, and cough). Diuretic drugs of medicinal plant origins without harmful effects can be considered a better alternative to commercial drugs. The angiotensin-converting-enzyme inhibitory (ACEI), diuretic, and hypotensive effects of the aqueous methanol crude extract of *M. emarginata* aerial parts administered intravenously were tested in Sprague-Dawley rats by the authors of [109]. Dimethyl sulphoxide and captopril served as the control and standard drugs, respectively, for the ACEI test. For the diuretic test, normal saline and frusemide (10 mL Kg^−1^, IP) served as control and standard drug, respectively. *M. emarginata* extract showed serum ACEI activity (IC_50_ value of 422 μg mL^−1^; 2.0 mg mL^−1^ extract had the highest (81%) ACEI effect compared with captopril (89%)). Additionally, the extract increased the volume of urine and excretion of urinary Na^+^ at 30 mg Kg^−1^ and 50 mg Kg^−1^ doses. The extract’s ACEI activity was ascribed to the presence of phytochemicals (like tannins, flavonoids, and alkaloids) via enzyme metal co-factor sequestration, protein precipitation, or other mechanisms. Similarly, the diuretic activity was ascribed to the phytochemical (such as alkaloids and phenolics) contents of *M. emarginata* extract. The extract exhibited a dose-dependent drop in the average arterial blood pressure range of 21.5–61.7% at a concentration range of 0.1–3.0 mg Kg^−1^. The authors alluded to vasodilation with heightened cardiac output to have led to a remarkable decrease in diastolic blood pressure. Additionally, it was reported that the plant extract caused heart rate decline, which was attributed to reflex mechanism involvement. Other mechanisms, such as indirect and/or direct effect on heart, inhibition, and/or relaxation of vascular smooth muscle contraction, leading to decreased total peripheral resistance or combination of mechanisms, were suggested.

In an in vivo study, the diuretic activity of the aqueous extract of *M. emarginata* leaves (200, 400, and 600 mg/kg b.w.) was investigated in adult female Wistar albino rats [79]. Normal drinking water (10 mL/kg b.w) and a commercial diuretic drug (furosemide 20mg/kg b.w) served as controls. The diuretic activity of the extract was confirmed by analyzing the disparity in the total volume of urine and diuretic markers, compared to the controls. *Merremia emarginata* leaf extract demonstrated a significantly higher diuretic effect in treated rats compared to the control group rats. This diuretic activity was without side effects such as proteinuria or glycosuria. Furthermore, a polyphenolic compound (chlorogenic acid) (**6**) was identified through ultra-performance liquid chromatography-tandem mass spectrometry (UPLC-MS/MS) and reverse phase-high performance liquid chromatography (RP-HPLC) to be responsible for the diuretic activity. These results suggest that *M. emarginata* may potentially act as a good diuretic agent without causing harmful side effects.

##### Wound Healing Property

Wounds result from physical injuries, that lead to a break or opening of the skin [110]. Proper wound healing is important to restore the disrupted functional status of the skin, as well as the interrupted anatomical continuity [110]. Several medicinal plants are used in traditional medicine to treat wounds because of their wound-healing properties [110,111]. Such medicinal plants promote wound healing by the activities of their bioactive substances (e.g., flavonoids, phenolic acids, tannins, etc.). The bioactive compounds in medicinal plants exhibit exceptional fast healing by reducing lipid peroxidation, thereby improving vascularity and preventing or slowing the onset of necrosis, and initiating skin cell differentiation [110,111]. The wound-healing activity of different ethanol extract fractions (solvent ether, petroleum ether, ethyl acetate, butanol, and butanone) of *M. tridentata* roots was tested in albino mice by Bidkar et al. [53] using the tensile strength of wound models (re-sutured incision and grass pith granuloma) on the 10th day after wounding. Orally administered Tween 80 solution served as the control. The results showed that all extract fractions (250 mg kg^−1^ or 300 mg kg^−1^) exhibited considerably higher tensile strength (215.4 g, 213 g, 243.7 g, 236.1 g, and 232.9 g, respectively) than the control (144.3 g) in the re-sutured incision model. Similarly, all extract fractions exhibited considerably higher tensile strength (221.7 g, 210 g, 264.1 g, 332.8 g, and 335.7 g, respectively) than the control (155.4 g) in the granuloma model. The wound-healing effect was attributed to the astringent property of flavonoid (present in the extracts), which allowed for wound contraction and enhanced epithelialization rate.

Sakinah et al. [112] fractionated *M. mammosa* ethanol extract and used 25 mg of ethyl acetate, n-hexane, and water fractions to evaluate the wound-healing properties of *M. mammosa* plant in diabetic male Wistar rats. Diabetes was induced in rats by administering streptozotocin (40 mg kg^−1^ BW, IP), while the wound was excised using the Morton method; aqua dest and gentamicin served as negative and positive controls, respectively. The ethyl acetate, n-hexane and water fractions of *M. mammosa* extract showed the smallest wound diameter (72%, 62%, and 54%, respectively) compared with the negative control (114 mm) on day 11. Additionally, *M. mammosa* extract fractions showed wound reduction (89%, 90%, and 93% in ethyl acetate, n-hexane, and water fractions, respectively) similar to the positive control (92%) and higher than the negative control (82%) on day 11. It was suggested that the anti-inflammatory effect of the flavonoid glycoside content of *M. mammosa* extract fractions might be responsible for the wound-healing property.

In another study, Marchianti et al. [57] investigated the wound-healing potency of 1.5% *M. mammosa* plant gel formulations (incorporation of hydroxypropylmethylcellulose (HPMC), Carbopol, or sodium carboxymethylcellulose (Na CMC) into 10% water fraction of the plant’s ethanol extract) in diabetic Wistar rats by percentage wound size reduction, vascular endothelial growth factor expression, hydroxyproline levels, and histopathology assessments. Diabetes was induced by streptozotocin (40 mg kg^−1^ BW, IP), while the wound was excised using the Morton method. The rats were divided into five treatment groups, viz. distilled water (negative control), neomycin sulfate and placenta extract gel (positive control), and 10% water fraction of *M. mammosa* extract in the gelling agents (3. HPMC, 4. Carbopol, and 5. Na CMC). All three *M. mammosa* gel formulations showed improved healing than the negative control and similar healing effect relative to the positive control in terms of optimum level vascular endothelial growth factor expression, hydroxyproline levels, and collagen density. The improved wound-healing effect of the three gel formulations was ascribed to the activities of flavonoid, as well as resin glycosides present in *M. mammosa* water fraction, restoring the delayed diabetic wound-healing process. The different investigations on the wound healing properties of *Merremia* species show that the extracts and fractions have potent wound healing abilities which support the traditional use of these species in treating wounds and inflammation.

##### Antioxidant Activity

Usually, the body keeps up a balance between the production of free radicals and their elimination. However, overproduction of reactive oxygen species leads to a disproportion between pro and antioxidants, resulting in oxidative stress [113]. Oxidative stress disrupts and damages DNA, cell structures, proteins, and lipids in the body and consequently results in neurodegenerative diseases, cancer, and diabetes, among others [113,114]. Antioxidants are useful chemical compounds that can reduce free radicals, decrease the rate of production, and even quench free radicals in the body [115]. Plants, including *Merremia* species, are rich sources of antioxidants. The antioxidant activities of the crude extracts (ethyl acetate, hexane, methanol, and 25% aqueous methanol) of *M. emarginata* whole plant were evaluated by 2,2-diphenyl-1-picrylhydrazyl (DPPH) and superoxide radical scavenging activity methods [116]. Vitamin C served as the antioxidant standard. Of the different extracts tested, the greatest potential scavenging activity was found to be the methanol crude extract (IC_50_ 8.6 µg mL^−1^). However, the scavenging activity of the reference drug (IC_50_ 3.3 µg mL^−1^) was higher than all extracts tested. The authors inferred that the phytochemical constituents of *M. emarginata* were responsible for the observed bioactivity. In another study, the antioxidant activity of 50–200 µg mL^−1^ solvent extracts (acetone, chloroform, methanol, and hot water) of *M. tridentata* roots and aerial parts were evaluated using DPPH, 2,2′-azino-bis (3-ethylbenzothiazoline-6-sulfonic acid) (ABTS), ferric reducing antioxidant power (FRAP), phosphomolybdenum reduction, ferrous ion (Fe^2+^) chelation, β-carotene/linoleic acid peroxidation inhibition, and antihemolytic activity assays [54]. Acetone root extract exhibited a higher antioxidant activity (IC_50_ 26.6 µg mL^−1^) than that of α-tocopherol standard in the DPPH assay. Compared to other extracts, *M. tridentata* acetone root extract exhibited the highest total antioxidant activity (26,270.8 µmol g^−1^) in the ABTS assay, highest ferric reducing antioxidant activity (2656.7 mmol Fe (II) mg^−1^) in the FRAP assay, and the strongest phosphomolybdenum reduction (56.7 g ascorbic acid/100 g) in the phosphomolybdenum reduction assay. The reducing power activity of the extracts was linked to high levels of phenolics. Also, the results showed a concentration-dependent OH^•^ scavenging activity (range: 29.6–59.3% and 34.8–52.9% by the root and aerial parts extracts at 200 µg mL^−1^). In the Fe^2+^ chelation assay, the hot water extract of *M. tridentata* aerial parts exhibited the highest activity (8.0 mg EDTA g^−1^ extracts). All the plant extracts inhibited β-carotene bleaching (range: 20.7–36.1% by the root extract and 13.3–32.9% by the aerial parts extract at 200 µg mL^−1^); acetone extracts (aerial parts and roots) and methanol root extract exhibited activities (32.9%, 36.1%, and 31.8%, respectively) comparable with BHA standard (36.6%). The distinct activity of *M. tridentata* acetone extract was suggested to be due to its high polyphenolics content. All the extracts showed higher peroxidation inhibition activity (aerial parts: 13.3–32.9%, roots: 20.7–36.1% at 200 µg mL^−1^) compared with α-tocopherol standard. The results of the antihemolytic activity assay showed that the acetone extract of *M. tridentata* roots was the most effective with 82.7% red blood cell hemolysis inhibition. The methanol and acetone extracts of the aerial parts showed comparable (70.5% and 74.0%, respectively) hemolysis inhibiting activity. The results of this study clearly show that the various solvent extracts of *M. tridentata* roots and aerial parts (particularly the acetone root extract) possess significant free radical scavenging and antioxidant activities. In another similar study, the antioxidant potency of different concentrations of aqueous extract of *M. emarginata* leaves was carried out in an in vitro study using reducing power, DPPH, ABTS, superoxide anion scavenging, and lipid peroxidation inhibition assays [21]. The authors also evaluated the leaf extract’s antioxidant activity based on fluorescence quenching using TGA-capped-CdTe QDs as fluorescent probes. The results showed that the reducing power of *M. emarginata* leaf extract increased proportionally to concentration. The extract showed optical density (0.79) comparable to butylated hydroxytoluene standard (1.32) at 1000 µg mL^−1^. The leaf extract’s reducing power was attributed to reductones’ presence, stabilizing and terminating radical chain reactions. The DPPH assay results showed that the leaf extract had antioxidant potency (IC_50_ value of 86.5 µg mL^−1^) relative to butylated hydroxytoluene standards (IC_50_ value of 23.4 µg mL^−1^), and the effect was ascribed to its antioxidant (e.g., flavonoids and polyphenolics) contents. In the ABTS assay, the extract (100 µg mL^−1^) showed ca. 71% inhibition (with IC_50_ value of 30.1 µg mL^−1^) relative to butylated hydroxytoluene standards (IC_50_ value of 27.2 µg mL^−1^). The leaf extract showed superoxide anion scavenging activity (IC_50_ value of 40.3 µg mL^−1^). The results of the TGA-capped-CdTe QD assay showed fluorescence emission quenching by *M. emarginata* extract. The quenching of fluorescence emission was attributed to the prevention of the electron-hole recombination process, causing a decrease in the fluorescence intensity due to the trapping of TGA-capped-CdTe QD holes by *M. emarginata* leaf extract.

Purushoth et al. [102] also subjected *M. emarginata* whole plant ethanol extract and its fractions (chloroform, ethyl acetate, hexane and methanol) to an in vitro antioxidant potency test using α, α-diphenyl-β-picrylhydrazyl (DPPH) scavenging, hydrogen peroxide scavenging, ABTS scavenging, and hydroxyl radical in the para-nitroso dimethylaniline assays. The DPPH assay results showed that the extracts had moderate to high antioxidant potency. However, the ethanol extract and methanol fraction had the highest antioxidant potency (IC_50_ values of 26.5 µg mL^−1^ and 27.5 µg mL^−1^, respectively). All extracts showed high antioxidant activity in the ABTS assay with IC_50_ values range of 15–38 µg mL^−1^ relative to ascorbic acid and rutin standards (IC_50_ values of 18.5 µg mL^−1^ and 12.7 µg mL^−1^, respectively). The antioxidant potency of the essential oil and different extracts (aqueous ethanol, butanol, chloroform, ethyl acetate, and hexane) of *M. borneensis* leaves and stems measured by reducing power (phosphomolybdenum method), β-carotene-linoleate model system, and DPPH radical scavenging assays revealed that all *M. borneensis* extracts showed antioxidant potency. However, the aqueous ethanol extracts showed the highest antioxidant potency in all the assays (with 31% antioxidant capacity in the phosphomolybdenum method, 84% inhibition of β-carotene bleaching, and 80% DPPH radical scavenging activity at 100 µg mL^−1^). It was suggested that antioxidant responses might be due to the quantity and/or variety of phenolics present in the leaf extracts of *M. borneensis* [22]. In another study by Joshi et al. [117]*,* the antioxidant potential of methanol extract of different parts (callus, leaf, seed and stem) of *M. dissecta* and *M. aegyptia* was evaluated using DPPH free radical scavenging assay; ascorbic acid was used as a standard. The study revealed an increasing free radical scavenging activity with increasing plant extracts concentration in both *Merremia* species. The leaf, seed, and stem extracts of *M. dissecta* had 75%, 90%, and 93% free radical scavenging effects with IC_50_ values of 82, 80 and 61 µg mL^−1^, respectively, while the seed extract of *M. aegyptia* had a free radical scavenging effect of 90% comparable to ascorbic acid with 92% at 500 μg mL^−1^. The authors attributed the observed antioxidant activity to the possible presence of other bioactive compounds like ascorbic acid, tocopherol, and pigments in the plants since the flavonoid contents did not correlate with observed antioxidant activity. To assess the pharmacological properties of a Thai traditional herbal formula (Sahatsatara), Thamsermsang et al. [118] used 2,2-dipheny-1-picrylhydrazyl (DPPH) assay to test the free radical scavenging activity of the ethanol extracts (3.75 μg mL^−1^, 15 μg mL^−1^, 30 μg mL^−1^, 60 μg mL^−1^, 120 μg mL^−1^) of its 21 ingredients, including *M. vitifolia* stem. Gallic acid, L-ascorbic acid and piperine were used as references. *M. vitifolia* showed a dose-dependent free radical scavenging ability, ranking fourth (with the IC_30_ value of 25 μg mL^−1^) relative to other components. The free radical scavenging activity of Sahatsatara was thus attributed to gallic acid and other unidentified bioactive compounds present in the formula’s components.

In a recent study, the antioxidant potency of 2 mg GAE mL^−1^ of hexane, ethyl acetate and methanol extracts of *M. mammosa* plant was tested using DPPH, hydroxyl radical and superoxide anion scavenging methods [56]. In the DPPH assay, *M. mammosa* methanol extract showed higher antioxidant activity relative to the positive control with IC_50_ values of 0.7 GAE ml^−1^ and 0.9 GAE ml^−1^, respectively. In the hydroxyl radical scavenging assays, *M. mammosa* methanol extract exhibited higher antioxidant activity relative to positive control with 92% and 89% inhibition values, respectively. The superoxide anion assay showed that *M. mammosa* methanol extract had higher antioxidant activity relative to hexane and ethyl acetate extracts (with inhibition values of 25%, 20%, and 19%, respectively). In a more recent study, the antioxidant activity of different extracts (N-Heksan, ethyl acetate, and methanol) of *M. peltata* leaf and stem was determined by DPPH radical scavenging and FRAP methods [90]. Ascorbic acid served as the standard antioxidant. Of all the sample extracts, the methanolic stem extract showed the best antioxidant activity (IC_50_ values 47.37 μg/mL) in the DPPH radical scavenging assay as well as the highest total antioxidant power (with value 207.08 μmol/g) in the FRAP assay. However, these activities were still lower than that of ascorbic acid with an IC_50_ value of 10.49 μg/mL in the DPPH assay and a total antioxidant power value of 340.04 μmol/g in the FRAP assay.

Collectively, the results from the different antioxidant potency evaluations of *Merremia* species indicate that the extracts are effective as natural antioxidants. None of the extracts tested demonstrated weak or inactive antiradical potential, rather the antioxidant activities displayed could be classified as either very strong, strong, and moderate. Antioxidant activity is classified into four groups based on IC_50_ values; very strong (<50 μg/mL), strong (50–100 µg/mL), moderate (101–250 µg/mL), weak (251–500 µg/mL) and inactive (˃500 µg/mL) [119]. Hence, *Merremia* species extracts can be utilized for use as dietary supplements or as functional ingredients in nutraceutical and pharmaceutical products.

##### Insecticidal Activity

The development of plant-based insect repellents that are effective, non-toxic, and have a short half-life will benefit both farmers and the environment by preventing the spread of vector-borne diseases [120,121]. The exploitation of bioactive constituents in plants with insecticidal activity are being considered as alternatives to chemical insecticides and pesticides as a result of their lower persistence in the environment, as well as low toxicity effects on non-target organisms [120,121]. Some *Merremia* species have been investigated for their insecticidal activities. Oliveira et al. [122] screened 94 extracts from 10 plants, including the extracts (250 µg mL^−1^ of acetone and its fractions (hexane, chloroform, ethyl acetate, and methanol)) of *M. aegyptia* leaves, for larvicidal activity against *Aedes aegypti* commonly found in the Northeast region of Brazil. Dimethyl sulfoxide (DMSO) and Temephos (a synthetic insecticide) served as the negative control and positive controls, respectively. It was reported that nine extracts, including acetone extract and the hexane fraction of *M. aegyptia* leaves exhibited over 100% activity (with LD_50_ values of 120.7 µg mL^−1^ and 144.3 µg mL^−1^, respectively) against the mosquitoes fourth instar larvae. The larvicidal activity was suggested to be due to the presence of bioactive compounds.

In another study, the larvicidal activity of aqueous extract (70 μg mL^−1^, 140 μg mL^−1^, 210 μg mL^−1^, 280 μg mL^−1^, and 350 μg mL^−1^) and silver nanoparticles (4 μg mL^−1^, 8 μg mL^−1^, 12 μg mL^−1^, 16 μg mL^−1^, and 20 μg mL^−1^) from *M. emarginata* leaves was tested on late third instar larvae of *Anopheles stephensi*, *Aedes aegypti* and *Culex quinquefasciatus* [123]. Distilled water and silver nitrate served as controls. It was reported that the aqueous leaf extracts and biosynthesized silver nanoparticles had a larvicidal effect on the mosquitoes in a dose-dependent manner. However, in comparison with the aqueous leaf extract of *M. emarginata,* the biosynthesized silver nanoparticles had higher larvicidal activity against *A. stephensi*, *A. aegypti* and *C. quinquefasciatus* larvae with LC_50_ values of 8.4 μg mL^−1^, 9.2 μg mL^−1^ and 10.0 μg mL^−1^, respectively at 20 μg mL^−1^. The *M. emarginata* biosynthesized silver nanoparticles were reported to be safer to non-target aquatic biocontrol agents (*Anisops bouvieri*, *Diplonychus indicus* and *Gambusia affinis*), with LC_50_ range of 416 μg mL^−1^ to 25,154 μg mL^−1^. It was suggested that the silver nanoparticles enhanced the leaf extracts’ bioactivity. The authors attributed the larvicidal activity to the silver nanoparticles and the mosquitoes’ larvae extracellular lipoprotein matrix interaction, increasing cell plasma membrane permeability. They further reported that the interaction(s) between the silver nanoparticles and phosphorous- or sulfur-containing compounds might have caused enzymes and organelles denaturation, thereby reducing ATP synthesis, which leads to the loss of cellular function and death. The low toxicity effect of *M. emarginata* silver nanoparticles on non-target organisms was suggested to be partially due to the bigger body size of organisms compared with mosquito instars.

A study was carried out by Santos et al. [80] on the control of coffee plants pest, *Leucoptera coffeella*, by oviposition reduction using methanol leaf extracts of 19 plants, including *M. tomentosa* (8.9 mg mL^−1^). The authors also assessed the effect of three isolated compounds (*cis*-tiliroside (**2**), *trans*-tiliroside, and ursolic acid (**59**)) from *M. tomentosa* leaves; Tween 80 and chlorpyrifos served as negative and positive controls. It was reported that only *M. tomentosa* methanol extract, ursolic acid (**59**) and *cis*-tiliroside (**2**) were effective in reducing *L. coffeella* oviposition on coffee plant leaves with oviposition reduction of 6%, 0% and 11%, respectively like the control chlorpyrifos (0%). Two of the isolated bioactive compounds (ursolic acid (**59**) and *cis*-tiliroside (**2**)) from *M. tomentosa* leaf extract were suggested to be responsible for the oviposition reduction by inhibiting glycogen phosphorylases (by binding to the pest’s allosteric site) and xanthine dehydrogenases, respectively. The results suggest a possible use of *Merremia* species extract in the production of green insecticides and repellents to control agricultural pests and mosquito vectors.

**Table 6:** Summary of the biological activities of *Merremia* species.

Species	Biological Activity	Plant Part	Extract	Concentration or Dose	Model	Result	Reference
*M. aegyptia*	Antioxidant activity	Seeds	Methanol	500 μg mL^−1^	In vitro: DPPH assay; ascorbic acid was used as a standard	Extract had 90% (IC_50_ values of 84) free radical scavenging effect	[117]
	Insecticidal activity	Leaves	Acetone and its hexane fraction	250 µg mL^−1^	In vivo: larvicidal activity against *Aedes aegypti* fourth instar larvae; Temephos served as a standard	Extracts exhibited over 100% activity (with LD_50_ values of 120.7 µg mL^−1^ and 144.3 µg mL^−1^, respectively)	[122]
*M. borneensis*	Antifungal	Leaves	Methanol	60–400 mg mL^−1^	*In vitro*: antifungal activity against a mold strain (*A. brasiliensis*) and two yeast strains (*Candida albicans* and *S. cerevisiae*) using agar well diffusion method	Extract showed concentration-dependent activity (inhibition zones range: 5.5–15.5 mm)	[63]
	Antioxidant activity	Leaves, stems	Aqueous ethanol	100 µg mL^−1^	In vitro: phosphomolybdenum method, β-carotene-linoleate model system and DPPH assays; ascorbic acid and BHA served as references	Phosphomolybdenum method: 31% antioxidant capacity, β-carotene bleaching: 84% inhibition, DPPH: 80% radical scavenging	[22]
*M. dissecta*	Antibacterial	Leaves, flowers	Diethyl ether, methanol	100 µg mL^−1^	In vitro: antibacterial activity against *S. aureus*, *P. aeruginosa* by biofilm inhibition; quorum sensing inhibitor (azithromycin) served as a control	Extracts decreased the biosynthesis of N-acyl homoserine lactone by 72%, attenuated the expression of elastase activity (27%) and biofilm production (55%) in *P. aeruginosa*	[4]
	Antioxidant activity	Leaves, seeds, stems	Methanol	500 μg mL^−1^	In vitro: DPPH assay; ascorbic acid was used as a standard	Leaves, seeds and stems extracts exhibited 75%, 90%, and 93%, free radical scavenging with IC_50_ of 82, 80, and 61 µg mL^−1^, respectively	[117]
*M*. *emarginata*	Cancer cell cytotoxicity	Whole plant	Ethyl acetate	50 μg mL^−1^	In vitro: human cancer cell lines (prostate carcinoma (DU-145); Lung carcinoma (A549), mouth carcinoma (KB) and pancreas carcinoma (MIA-PaCa-2)) using the 3-(4,5-Dimethylthiazol-2-yl)-2,5-diphenyltetrazolium bromide (MTT) reduction assay	Ethyl acetate extract was the most effective, inhibiting the proliferation of cell lines A549, KB, MIA-PaCa-2, and DU-145 with the IC_50_ values of 28.5 μg mL^−1^, 37.2 μg mL^−1^, 51 μg mL^−1^, and 69.4 μg mL^−1^, respectively	[3]
	Cancer cell cytotoxicity	Leaves	Hexane	25 μg mL^−1^	In vitro: human cancer cell lines (lung (A549), breast (MCF-7), stomach (AGS), colon (COLO 320 DM)) and monkey normal kidney epithelial cells (VERO)	Extract inhibited A549 and COLO 320 DM cell line proliferation (with IC_50_ values of 15.5 μg mL^−1^ and 18.4 μg mL^−1^, respectively) and had minimal toxicity (IC_50_ value of 65.2 μg mL^−1^) for VERO	[88]
	Anti-diabetic	Whole plant	Methanol	100, 200, and 400 mg kg^−1^	*In vivo*: streptozotocin-induced (intraperitoneally) diabetic male Wistar rats; assessment of blood glucose levels, plasma insulin and body weight, glycosylated hemoglobin, total hemoglobin, serum creatinine, serum urea, hexokinase, fructose-1, 6-bisphosphatase and glucose-6-phosphatase, total protein and pancreatic tissue histology	All extract doses lowered blood glucose levels to values comparable to standard drug (glibenclamide) and restored plasma insulin, body weight, glycosylated hemoglobin, total hemoglobin, serum creatinine, serum urea, hexokinase, fructose-1, 6-bisphosphatase, and glucose-6-phosphatase, and total protein to levels near normal control (sodium chloride). In the histological assessment, the extracts (100 and 200 mg kg^−1^, respectively) showed mild and moderate expansion of the diabetic rats’ pancreatic islets, while the 400 mg kg^−1^ extract showed prominent hyperplastic islet similar to that of the glibenclamide-treated group	[93]
	Antibacterial	Leaves	Aqueous, petroleum ether, and methanol	10 µg mL^−1^, 25 µg mL^−1^, 50 µg mL^−1^, 100 µg mL^−1^	In vitro: antibacterial activity against *B. cereus*, *E. coli*, *P. aeruginosa*, and *S. aureus* by disk diffusion assay; penicillin standard drug	All solvent extracts were effective against all the bacteria (zone of inhibition range: 0.7–13.7 mm) compared with penicillin (16.0–17.0 mm)	[95]
	Antibacterial	Leaves	Aqueous, acetone, chloroform, petroleum ether	200 mg mL^−1^	In vitro: antibacterial activity against seven bacteria by paper disk diffusion; Ciprofloxacin as a reference drug	All solvent extracts were effective against five bacteria, particularly *L. rhamnosus* (20% zone of inhibition each); the most effective (acetone extract) had maximum inhibitory (24%) against *S*. *epidermidis*	[96]
	Antibacterial	Leaves	Aqueous, thioglycolic acid-capped cadmium telluride quantum dots, their mixture (T-M complex)	1 × 10^−6^ M, 100 µg	In vitro: antibacterial activity against *E. coli* using the Luria-Bertani agar disk diffusion assay	Extract alone and T-M complex showed 10-mm and 16-mm zones of inhibition, respectively	[21]
	Anti-inflammatory	Whole plant	Ethyl acetate	50 μg mL^−1^	In vitro: inhibition of pro-inflammatory cytokine, tumor necrosis factor alpha (TNF-α); recombinant human TNF-α served as standard	Extract inhibited TNF-α production with the IC_50_ of 5.9 μg mL^−1^.	[3]
	Anti-arthritic	Whole plant	Ethanol extract and its methanol and ethyl acetate fractions	250 μg mL^−1^	In vitro: anti-arthritic activity by protein denaturation inhibition method; diclofenac sodium served as a reference	Extracts had protein denaturation inhibition of 95.4%, 87.7%, and 72.6%, respectively, relative to the standard (98.4%)	[102]
	Antioxidant activity	Whole plant	Methanol	300 μg mL^−1^	In vitro: DPPH and superoxide radical scavenging assays; Vitamin C served as a standard drug	IC_50_ 8.6 µg mL^−1^ relative to vitamin C (IC_50_ 3.3 µg mL^−1^)	[116]
	Antioxidant activity	Leaves	Aqueous	100 µg mL^−1^ or 1000 µg mL^−1^	In vitro: reducing power, DPPH, ABTS, superoxide anion scavenging, and lipid peroxidation inhibition assays, fluorescence quenching using TGA-capped-CdTe QDs as fluorescent probes; butylated hydroxytoluene served as standards	Reducing power assay: optical density (0.79) comparable to butylated hydroxytoluene standard (1.32); DPPH assay: IC_50_ of 86.5 µg mL^−1^; ABTS assay: 71% inhibition with IC50 of 30.1 µg mL^−1^; superoxide anion scavenging activity: IC_50_ of 40.3 µg mL^−1^; TGA-capped-CdTe QD assay showed fluorescence emission quenching	[21]
	Antioxidant activity	Whole plant	Ethanol extract and its methanol fraction		In vitro: DPPH, hydrogen peroxide scavenging, ABTS scavenging, and hydroxy radical in the para-nitroso dimethylaniline assays; ascorbic acid, rutin, and BHA served as standards	DPPH assay: ethanol extract and its methanol fraction had IC_50_ of 26.5 µg mL^−1^ and 27.5 µg mL^−1^, respectively), ABTS assay: all extracts showed IC_50_ range of 15–38 µg mL^−1^	[102]
	Anti-urolithiatic activity	Whole plant	Methanol	400 µg mL^−1^	In vitro: anti-urolithiatic activity using a dissolution model; Cystone^®^ served as a positive control	Extract had calcium phosphate and calcium oxalate mineralization inhibition of 81% and 84%, respectively, relative to the standard drug (91%)	[107]
	Blood pressure-lowering property	Aerial parts	Aqueous methanol	30 mg Kg^−1^, 50 mg Kg^−1^; 0.1–3.0 mg Kg^−1^	In vivo: angiotensin-converting-enzyme inhibitory (ACEI), diuretic and hypotensive effects in Sprague-Dawley rats; captopril and frusemide served as standard drugs, respectively	Extract exhibited 81% ACEI activity, increased the volume of urine and excretion of urinary Na^+^, drop in average arterial blood pressure range of 21.5–61.7%	[109]
	Insecticidal activity	Leaves	Leaf silver nanoparticles	20 μg mL^−1^	In vivo: larvicidal activity against late third instar larvae of *A. stephensi*, *A. aegypti* and *C. quinquefasciatus*; distilled water and silver nitrate served as a control	Larvicidal activity on *A. stephensi*, *A. aegypti* and *C. quinquefasciatus* larvae with LC_50_ values of 8.4 μg mL^−1^, 9.2 μg mL^−1^, and 10.0 μg mL^−1^, respectively	[123]
	Nephroprotective activity	Leaves	Ethanol	250 mg kg^−1^	In vivo: nephroprotective activity against gentamicin-induced (intraperitoneally) nephrotoxicity in adult albino Wistar rats using a histopathology examination	Extract caused regeneration of glomerular, tubular, and proximal tubular epithelial cells of damaged kidney	[64]
*M. hederacea*	Multidrug resistance reversal activity	Aerial parts	Isolated compounds (merremins A−G and murucoidin IV, murucoidin V, stoloniferin IV, and murucoidin XVII)	25 μM	In vivo: multidrug resistance reversal activity in KB/VCR cell lines using a sulforhodamine B assay; vinblastine served as a reference	Noncytotoxic inhibition ratios less than 50% (0.91%, −0.08%. 0.23%. 7.73%, respectively at 25 µM) for compounds A, E, F, and murucoidin V with IC_50_ values of 0.253, 0.036, 0.230, 0.004, and 0.570	[74]
*M. mammosa*	Anti-diabetic	Whole plant	Hexane, ethyl acetate, methanol	25 µg GAE mL^−1^, 100 µL	In vitro: α-amylase and α-glucosidase inhibition	Extracts exhibited α-amylase inhibition (48%, 43%, and 12%, respectively) and α-glucosidase inhibition (66%, 52%, and 13%, respectively)	[56]
	Anti-influenza	Tuber	Ethyl acetate fraction of methanol extract	1000 μg mL^−1^	In vivo: anti-influenza A (subtype H1N1) activity using hemagglutinin assay; Zanamivir served as positive control	Extract had a strong anti-influenza effect similar to Zanamivir, reducing hemagglutinin virus titer by 94% and 100%, respectively, at 1000 μg mL^−1^	[99]
	Antioxidant activity	Whole plant	Methanol	2 mg GAE mL^−1^	In vitro: DPPH, hydroxyl radical, and superoxide anion scavenging; methanol and vitamin C served as negative and positive controls, respectively	Methanol extract showed IC_50_ value of 0.7 GAE ml^−1^ relative to the positive control (0.9 GAE ml^−1^) in the DPPH assay, 92% inhibition in the hydroxyl radical scavenging assays, 25% inhibition in the superoxide anion assay	[56]
	Wound-healing property	Whole plant	Ethyl acetate, n-hexane, and water fractions of ethanol	25 mg kg^−1^	In vivo: wound-healing effect in streptozotocin-induced (intraperitoneally) diabetic male Winstar rats; wound was excised using the Morton method; aquadest and gentamicin served as negative and positive controls, respectively	Extracts showed wound diameter (72%, 62%, and 54%, respectively) compared with the negative control (114 mm), and percentage wound reduction (89%, 90%, and 93%, respectively) similar to the positive control (92%)	[112]
	Wound-healing property	Whole plant	Gel formulations (hydroxypropylmethylcellulose (HPMC), Carbopol or sodium carboxymethylcellulose (Na CMC) in 10% water fraction) of ethanol extract	1.5% gelling agent	In vivo: vascular endothelial growth factor, hydroxyproline levels, and collagen density assessments in streptozotocin-induced (intraperitoneally) diabetic Winstar rats; wound was excised using the Morton method; distilled water (negative control), neomycin sulfate, and placenta extract gel (positive control)	Extracts had similar healing effect relative to the positive control in terms of optimum level vascular endothelial growth factor expression, hydroxyproline levels, and collagen density	[57]
*M. peltata*	Anti-diabetic	Leaves and stem	Hexane, ethyl acetate, and methanol	20-100 µg mL^−1^,	In vitro: alpha glucosidase enzyme inhibition	Stem methanolic extract had the best activity with IC_50_ value 47.44 μg/mL, almost two times better than acarbose as a positive control (IC_50_ = 98.38 μg/mL). Leaves methanolic extract, leaves ethyl acetate extract, and stem ethyl acetate extract also give better activity of alpha glucosidase inhibitors than acarbose with IC_50_ value 67.24 μg/mL, 69.38 μg/mL, and 72.85 μg/mL, respectively.	[90]
	Antibacterial	Leaves	Ethanol	5–20 µg mL^−1^	In vitro: antibacterial activity against *B. subtilis*, *S. aureus*, *P. aeruginosa*, *E. coli* using the Kirby-Bauer disk diffusion method; streptomycin and chloramphenicol served as positive controls	Extract showed 5.7-mm average zone of inhibition against *B. subtilis* and *S. aureus* (at 10 µg mL^−1^ and 20 µg mL^−1^, respectively), 4.7-mm and 2.7-mm average zones of inhibition against *P. aeruginosa* and *E. coli* (at 15 µg mL^−1^ and 5 µg mL^−1^, respectively)	[19]
	Anti-diabetic	Leaves and stem	Hexane, ethyl acetate, and methanol	20-100 µg mL^−1^,	In vitro: DPPH and FRAP	Stem methanolic extract had the highest antioxidant activity with IC_50_ value of 47.41 μg/mL in DPPH and total antioxidant power of 340.04 μmol/g in FRAP.	[90]
*M. tomentosa*	Insecticidal activity	Leaves	Methanol and isolated compounds (ursolic acid and cis-tiliroside)	8.9 mg mL^−1^	In vivo: oviposition reduction in *L. coffeella*; chlorpyrifos serves as positive control	Extract and isolated compounds reduced *L. coffeella* oviposition to 6%, 0%, and 11%, respectively) relative to the control chlorpyrifos (0%)	[80]
*M. tridentata*	Anti-diabetic	Roots	Aqueous	50–150 mg kg^−1^	In vivo: normoglycemic, glucose-loaded hyperglycemic, and streptozotocin-induced (intraperitoneally) diabetic rats; blood glucose levels, serum insulin, triglycerides, total cholesterol, glycogen (in skeletal muscle and liver), and lipid peroxidation in pancreatic tissue estimations	All extract doses lowered blood glucose levels in normoglycemic, glucose-loaded hyperglycemic and streptozotocin-induced diabetic rats (range: 13.4–26.2%, 13.0–20.1%, and 28.6–49.7%), respectively) compared with their control groups; glibenclamide (reference drug) reduced blood glucose levels by 61.7%; in streptozotocin-induced diabetic rats, all extract doses increased serum insulin levels (maximum increase (14.9 U mL^−1^) was comparable to glibenclamide (18.3 U mL^−1^) at 150 mg kg^−1^; dose-dependent reduction (range: 13.9–25.1%) of serum triglycerides reduction; 100 mg kg^−1^ and 150 mg kg^−1^ extracts lowered serum cholesterol levels (ca. 60 mg dL^−1^) compared with both normal (66 mg dL^−1^) and diabetic (139 mg dL^−1^) controls, increased glycogen levels compared with streptozotocin-induced diabetic control and reduced lipid peroxidation levels (0.46 µM g^−1^, 0.47 µM g^−1^) to near glibenclamide (0.41 µM g^−1^)	[52]
	Antibacterial	Leaves	Methanol	0.01 mg mL^−1^, −1.00 mg mL^−1^;0.05 mg mL^−1^, −1 mg mL^−1^	In vitro: antibacterial activity against Gram-positive (*Bacillus subtilis* and *Staphylococcus aureus*) and Gram-negative (*Escherichia coli* and *Vibrio parahaemolyticus*) bacteria using turbidity and zone formation methods	Gram-positive MIC range: 0.25–0.5 mg mL^−1^; zone formation range: 10‒14 mm; Gram-negative MIC range: 0.5–1 mg mL^−1^; zone formation range: 9–11 mm	[94]
	Antibacterial	Stem	Methanol	100 mg mL^−1^	In vitro: antibacterial activity against *S. aureus* and *B. subtilis*, *Klebsiella pneumoniae*, *E. coli*, and *Pseudomonas aeruginosa*; ampicillin and gentamicin standard drugs	Extract was effective against *S. aureus* and *B. subtilis* (inhibition zones: 12.0 mm and 11.3 mm; MIC: 3.13 mg mL^−1^ and 6.25 mg mL^−1^, respectively; minimum bactericidal concentration: 12.5 mg mL^−1^ and 100 mg mL^−1^, respectively) relative to both standard drugs (10 μg mL^−1^	[66]
	Antifungal	Leaves	Methanol	0.01–100 mg mL^−1^	In vitro: antifungal activity against *Aspergillus niger*, *Saccharomyces cerevisiae* using turbidity and zone formation methods	Extracts were more effective against *S. cerevisiae* (MIC range: 0.25–0.5 mg mL^−1^; zone formation range: 10–15 mm).	[94]
	Anti-inflammatory	Whole plant	Ethanol	100 mg kg^−1^, 200 mg kg^−1^	In vivo: anti-inflammatory activity on male albino rats using the carrageenan-induced rat paw inflammation method; indomethacin served as standard drug	Relative to the standard drug (48.5%), extract was effective with 38.3% and 42.8% inflammation inhibition at 100 mg kg^−1^ and 200 mg kg^−1^ doses, respectively	[55]
	Anti-inflammatory	Roots	Solvent ether, ethyl acetate, butanol and butanone fractions of ethanol extract	300 mg kg^−1^	In vivo: anti-inflammatory activity in albino mice using the carrageenan-induced rat paw edema method; aspirin served as the standard drug	Extracts were effective (87%, 84%, 72%, and 68%, respectively) in inhibiting rat paw edema, relative to the standard (59.7%)	[53]
	Anti-arthritic	Whole plant	Ethanol	100 mg kg^−1^ and 200 mg kg^−1^	In vivo: anti-arthritic activity on male albino rats using the complete Freund’s adjuvant-induced arthritis model; indomethacin served as the standard drug	Extract had 49.0% and 51.7% anti-arthritic activity at 100 mg kg^−1^ and 200 mg kg^−1^, respectively, which were comparable to the standard drug (55.5%)	[55]
	Antioxidant activity	Roots, aerial parts	Acetone, chloroform, methanol, and hot water	200 µg mL^−1^	In vitro: DPPH and ABTS, OH^•^, FRAP, phosphomolybdenum reduction, Fe^2+^ chelation, β-carotene/linoleic acid peroxidation inhibition, and antihemolytic activity assays	Acetone root extract: IC_50_ 26.6 µg mL^−1^ in DPPH assay; highest equivalent trolox, FRAP and phosphomolybdenum reduction values (26,270.8 µmol g^−1^ extract, 2656.7 mmol Fe (II) mg^−1^ extract and 56.7 g ascorbic acid/100 g extract, respectively); root and aerial parts extracts: OH^•^ scavenging activity range of 29.6–59.3% and 34.8–52.9%; hot water extract showed activity of 8.0 mg EDTA g^−1^ extracts in the Fe^2+^ chelation assay; inhibition of β-carotene bleaching (20.7–36.1% by the root extract and 13.3–32.9% by the aerial parts extract); acetone extracts (aerial parts and roots) and methanol root extract exhibited activities (32.9%, 36.1%, and 31.8%, respectively) comparable with BHA standard (36.6%); higher peroxidation inhibition activity by all extracts compared with α-tocopherol standard; acetone root extract: 82.7% red blood cell hemolysis inhibition	[54]
	Wound-healing property	Roots	Solvent ether, petroleum ether, ethyl acetate, butanol, and butanone fractions of ethanol	250 mg kg^−1^ or 300 mg kg^−1^	In vivo: wound-healing effect in albino mice using the tensile strength of wound models (re-sutured incision and grass pith granuloma); Tween 80 solution served as the control	Extracts exhibited higher tensile strength (215.4 g, 213 g, 243.7 g, 236.1 g, and 232.9 g, respectively) than the control (144.3 g) in the re-sutured incision model; (221.7 g, 210 g, 264.1 g, 332.8 g, and 335.7 g, respectively) than the control (155.4 g) in the granuloma model	[53]
*M. umbellata*	Antibacterial	Leaves	Ethanol	1000 μg mL^−1^	In vitro: antibacterial activity against *Klebsiella pneumoniae*, *S. aureus*, and *Pseudomonas aeruginosa* using the broth microdilution method; gentamicin was the positive control	Extract had 32%, 55%, and 67%, respectively, on the bacterial strains’ growth	[62]
*M. vitifolia*	Anti-arthritic	Leaves	Aqueous fraction from methanol extract	500 μg mL^−1^	In vitro: anti-arthritic activity by protein denaturation inhibition method; diclofenac sodium served as a reference	Extracts had protein denaturation inhibition of 64% relative the standard drug (87%)	[20]
	Anti-nociceptive activity	Leaves	Aqueous fraction from methanol extract	200 mg kg^−1^, 400 mg kg^−1^	In vivo: anti-nociceptive activity in Swiss Albino mice using formalin-induced paw licking (at early and late stages) and acetic acid-induced writhing tests; diclofenac sodium (administered intraperitoneally) served as a standard drug	Extract showed anti-nociceptive activity (44% and 26%, 30% and 20% in both early and late test stages, respectively) relative to the control (20% and 15%, respectively) in the formalin-induced paw licking test; reduced (44% and 30%, respectively) abdominal contortions with increasing doses of APFME relative to the control (21%)	[20]
	Antioxidant activity	Stems	Ethanol	120 μg mL^−1^	In vitro: DPPH assay; gallic acid, L-ascorbic acid, and piperine were used as references	IC_30_ of 25 μg mL^−1^	[118]
	Thrombolytic activity	Leaves	Aqueous fraction from methanol extract	500 μg mL^−1^	In vitro: thrombolytic activity in blood samples from male and female (1:1) adult human volunteers with no anticoagulant and oral contraceptive treatments history; streptokinase served as a positive control	Extract exhibited thrombolytic activity (42.5% clot lysis) relative to the positive control (72.2%)	[20]

Results are shown for the most effective doses.

## 4. Toxicology

It is important to evaluate the safety of species in the genus *Merremia* due to their traditional medicinal usage, and as feed for livestock, even though several species in the genus are non-toxic. However, only a few toxicity studies are available on *Merremia* species. Acute toxicity study of the ethanolic root extract of *M. tridentata* was performed at dose concentrations of 500 and 1000 mg/kg body weight. The extract was orally administered to Wistar albino rats and observed for mortality after 72 h. No visible toxicity, behavioral changes, or mortality were observed in rats [124]. In another study, an acute toxicity study of the methanolic whole plant extract of *M. emarginata* was evaluated in five groups of adult male Wistar rats. The whole plant extract was orally administered to the mice at dose levels of 100, 500, 1000, 2000, and 4000 mg/kg and observed for neurological, behavioral, and autonomic reactions after 24 h and 72 h. There was no toxic reaction and lethality in the experimental rats at any dose given until the end of the study period [93]. Similarly, Priya et al. [125] reported no toxic effect of the ethanolic leaf extract of *M. emarginata* at a dose of 2g/kg administered orally to Swiss albino mice after 24 h. A few *Merremia* species are considered toxic to ruminants. For example, spontaneous poisoning in cattle by *M. macrocalyx* in the Pernambuco state, north-eastern Brazil was reported by Brito et al. [126]. In a recent study, acute and chronic toxicity evaluation of the ethanolic leaf extract of *M. tridentata* was carried out on albino male rats [127]. In the acute toxicity study, animals were orally administered different extract doses of *M. tridentata* (10, 100, and 1000 mg/kg b.w.) and monitored for 24 h for toxicity signs. In the chronic toxicity study, the animals were orally administered different *M. tridentata* extract doses once a day (100, 200, 400 mg/kg b.w.) for 14 weeks (100 days). The control group received 0.2 mL of distilled water. Haematology, serum biochemical indices, and histopathological studies of the kidney, liver, heart, spleen, and lungs of the animals were carried out for the toxic effect of the extract. The results of the acute toxicity study revealed that there was no mortality of any of the rats at 10, 100, and 1000 mg/kg b.w. dose while mortality was only recorded at 5000 mg/kg b.w. dose. The median lethal dose was estimated to be 2200 mg/kg b.w. In the chronic toxicity study, the results showed that the extract did not cause any alteration in the hematological parameters at all doses when compared with the control. However, the extract at 400 mg/kg b.w. caused a significant reduction in the lymphocyte. Chronic exposure of the animals to the plant extract at 100 mg/kg dosage, when tested for biochemical parameters, had no significant difference when compared with the control animal. However, at higher dosages of 200 and 400 mg/kg for 100 days, there was a significant increase in the serum levels of the biochemical parameters investigated when compared with the control. The abnormal increase in values of the biochemical parameters such as creatine, urea, transaminases, creatine kinase, among others are indications of the potential toxicity of the extract to the kidney, liver, and heart. Further histopathological studies revealed that the extract at 200 mg/kg did not show any morphological sign of toxicity on histoarchitecture of the liver, spleen, kidney, lung, and heart, but caused hemorrhage and vascular congestion in the heart, kidney, and lungs. Also, the extract at 400 mg/kg resulted in renal, myocardial damage, fibrosis, and vascular congestion in the lung, spleen, and liver.

Although the few documented experimental toxicology studies revealed that most *Merremia* species are safe, the *Merremia* species extract at chronic administration even at a low dosage of 200 mg/kg b.w. may not be safe as inferred from the recent toxicity study. There is a need for more toxicity studies to be conducted on the different species to establish their safety, especially for modern clinical use. 

## 5. Discussion and Future Perspective

The current review presented the research progression in the genus *Merremia* and summarized knowledge on its nutritional value, ethnomedicinal uses, phytochemistry, biological activities, and toxicity studies. 

The bibliometric analysis of the genus *Merremia* evaluated the global research trends between 1990 and 2020 based on the relevant data retrieved from Web of Science. We found that a high number of research articles (55%) were published in the last decade (2011–2020). This may be due to the availability of funds majorly because of the wide traditional uses and bioactivities, the advent of research ideas, and sophisticated analytical tools for chemical analysis. Moreover, interest has been rising for natural product research and development across the globe [128]. The utilization of medicinal plants (particularly from commonly used medicinal plants) as alternative sources for identifying bioactive agents that pharmaceutical industries can employ in the preparation of drugs in modern medicine has substantially risen [129]. These suggest that there may be more funding opportunities for *Merremia* research, which in turn suggests that articles related to the *Merremia* species are likely to increase in years to come.

Most of the leading authors on *Merremia* related research based on continental production were from Asia. This correlates with the fact that most *Merremia* species are primarily distributed in the warm and tropical regions of Asia [74]. As identified from the bibliometric analysis, the current research on the *Merremia* species can be grouped into four thematic areas, viz. drug formulation, chemical analysis (including the nutritional value), treatment of diseases, and taxonomy. 

Based on the available reports on the nutritional and antinutritional constituents of *Merremia* species, they contain important nutritional components that are beneficial to humans. Additionally, the *Merremia* species can be considered good fodder as several evaluated species meet high nutritional quality criteria for animals, and are therefore suitable for inclusion in the diets of livestock. However, the nutritional analysis and toxicity testing of *Merremia* species are still less researched as inferred from this study. Future investigation on this genus should include more nutritional and toxicity analyses of the species. 

Given the wide distribution of *Merremia* species in the Asia continent, the literature review revealed that members of the genus are mainly used ethnomedicinally in India, China, Indonesia, Malaysia, and the Philippines. Phytochemistry studies have resulted in the isolation of several secondary metabolites, including flavonoids, phenolics, sesquiterpenoid, alkaloids, resin glycosides, among others in the genus. Several pharmacological investigations have been carried out using in vivo and in vitro techniques in the genus. 

In terms of current research, several biological activities have been documented from different species of *Merremia*, including cancer cell cytotoxicity, antidiabetic, antimicrobial, anti-inflammatory, blood pressure-lowering, wound-healing, multi-drug resistance reversal activities, etc., which supports some of the ethnomedicinal uses of *Merremia* species. For example, leaves of *M. mammosa* and the root of *M. tridentata* have been used in the traditional treatment of diabetes and diabetic ulcer in Indonesia and India. Available studies have attributed the in vitro and in vivo antidiabetic and wound-healing properties in *M. mammosa* and *M. tridentata* to flavonoid and glycoside contents of the species. Also, extracts and fractionated extracts of *Merremia* species revealed that flavonoids are responsible for the anti-inflammatory activities, which supports the ethnomedicinal use of several *Merremia* species in treating inflammation and rheumatism.

In India, the Philippines, and Colombia, leaves and whole plants of *M. peltata*, *M. umbellata* and *M. emarginata* have been used traditionally as antibacterial and antifungal agents. In vitro antimicrobial studies in these species also showed effective antimicrobial activities that were attributed to different phytochemicals (alkaloids, flavonoids, leucoanthocyanidins, steroids, and phenolics) present in the species. 

Generally, most of the biological activities investigated in the *Merremia* species showed relevant and good activities with IC_50_ values below 100 µg mL^−1^. The end-point criteria set for medicinal plants to be considered as showing relevant bioactivity include; plant extracts with IC_50_ values below 100 µg mL^−1^ and pure compounds isolated from plants showing activities with IC_50_ values below 25 µM [1].

Several aspects highlighted below need to be considered and investigated further in the genus. (1) So far, only 11 species have been investigated for their bioactivities from a large genus of over 70 species. Based on the reported bioactivities in the investigated species, other uninvestigated species may be viewed as promising plant species with pharmacological importance. Most of the species that have not been investigated are native to Africa. However, Africa lags behind Asia, Australia, North, and South America in terms of research (including medicinal plant research), presumably due to limited funding resources and infrastructure. More funding should be made available to investigate the several under-exploited medicinal plants in Africa, including the *Merremia* species, as they offer untold bioactivities that are of nutritional and pharmaceutical relevance. Thus, future research should consider in vitro and in vivo evaluation of other species in this genus to discover new sources of phytochemicals and bioactivities that may be of pharmaceutical importance.

(2) Several species in the genus are used as traditional medicine to treat various ailments. However, only a few ethnomedicinal applications have been confirmed by scientific studies. Future studies need to be conducted to investigate and verify other ethnomedicinal applications. For example, studies evaluating the anticancer activity of *Merremia species* are limited in depth and scale. Future studies need to evaluate the cancer cell cytotoxicity activity of different species in a variety of animal models. Additionally, their ethnomedicinal uses in the treatment of stroke and also as alopecia should be investigated in scientific studies. 

(3) There are no reported clinical studies to assess the mechanism of action, efficacy, and safety of reported bioactivities in the genus. Clinical studies would be indispensable to adequately characterize these aspects.

(4) More studies are also needed to determine the exact phyto-compound responsible for the different bioactivities and discuss the relation between mechanisms and compounds. 

(5) Although most toxicity studies reported that *Merremia* species are safe to use, a recent toxicity study reported that chronic administration of *M. tridentata* for a prolonged period may cause damage to vital organs in the body [127]. Studies on toxicology and side effects are indispensable in the study of medicinal plants to determine their safety and optimal dose. More toxicological studies should also be the focus of future research in this genus. 

Overall, based on the reports of investigations in the genus, it can be concluded that species in the genus *Merremia* are a valuable nutritional and medicinal resource with promising therapeutic properties. Although the use of *Merremia* species as food or medicinal plants is encouraged, the long-term sustainability of the plants should also be considered, and as such destructive harvesting involving whole plants for therapeutic purposes should be discouraged to ensure conservation and sustainable use. Conclusively, future research should focus on comprehensive pharmacokinetics studies, in vivo studies, human clinical studies, and toxicity studies. These will show the clinical efficacy and support their development as therapeutic agents against several diseases.

## Figures and Tables

**Table 1:** Summarized bibliographic data.

Bibliographic Information	Numerical Outputs
Publications	357
Sources (Journals, Books, etc.)	188
Keywords Plus (ID)	2727
Author’s Keywords (DE)	1005
Period	1990–2020
Average citations per document	10.39
Authors	948
Author Appearances	1537
Authors of multi-authored documents	937
Single-authored documents	18
Average documents per author	0.377
Average authors per document	2.66
Average co-authors per document	4.31
Average collaboration index	2.76

**Table 2:** Top 20 authors’ countries on studies related to the genus—*Merremia* from 1990–2020. SCP—single country publications. MCP—multiple country publications. MCP/P—multiple country publications per publication.

Most Productive Countries								Total Number of Citations per Country			
Rank	Countries	Articles	% of Total	Freq	SCP	MCP	MCP/P Ratio	Rank	Country	Article Citations	Citation Average
1	Brazil	116	32.5	0.34	112	4	0.03	1	India	769	15.1
2	India	51	14.3	0.15	49	2	0.04	2	Brazil	697	6.0
3	Japan	27	7.6	0.08	26	1	0.04	3	Japan	536	19.9
4	USA	26	7.3	0.08	19	7	0.27	4	Germany	399	30.7
5	China	18	5.0	0.05	16	2	0.11	5	USA	295	11.4
6	Germany	13	3.6	0.04	10	3	0.23	6	Mexico	173	17.3
7	Indonesia	13	3.6	0.04	13	0	0.00	7	Malaysia	117	29.3
8	Mexico	10	2.8	0.03	8	2	0.20	8	China	102	5.7
9	Belgium	6	1.7	0.02	3	3	0.50	9	Panama	89	22.3
10	Venezuela	6	1.7	0.02	5	1	0.17	10	Spain	77	25.7
11	Australia	5	1.4	0.01	4	1	0.20	11	Colombia	67	22.3
12	South Africa	5	1.4	0.01	5	0	0.00	12	South Africa	53	10.6
13	Malaysia	4	1.1	0.01	4	0	0.00	13	Canada	41	20.5
14	Panama	4	1.1	0.01	2	2	0.50	14	Australia	40	8.0
15	Argentina	3	0.8	0.01	2	1	0.33	15	Argentina	36	12.0
16	Colombia	3	0.8	0.01	1	2	0.67	16	Belgium	31	5.2
17	Spain	3	0.8	0.01	1	2	0.67	17	Indonesia	28	2.2
18	Thailand	3	0.8	0.01	3	0	0.00	18	Venezuela	26	4.3
19	United Kingdom	3	0.8	0.01	1	2	0.67	19	Togo	22	22.0
20	Bangladesh	2	0.6	0.01	2	0	0.00	20	Thailand	17	5.7

**Table 3:** Taxonomic classification of the genus *Merremia*.

**Domain**	Eukaryota
**Kingdom**	Plantae
**Phylum**	Spermatophyta
**Subphylum**	Angiospermae
**Class**	Dicotyledonae
**Order**	Solanales
**Family**	Convolvulaceae
**Genus**	*Merremia*

**Table 4:** Ethnomedicinal uses of *Merremia* species.

Species	Ethnomedicinal Uses	Part Used	Type of Extraction	Country	Location of Collection	Reference
*M. borneensis*	Relieve breast cancer	Leaf	Maceration	Malaysia	Unspecified	[22]
	Hair treatment	Leaf	Maceration	Brunei Darussalam	Madang	[63]
	Relieve breast cancer	Aerial parts	Maceration	Malaysia	University of Malaysia, Sabah area	[59]
*M. emarginata*	Rheumatism, neuralgia, cough, and headache	Leaf	Maceration	India	Dharmapuri, Tamil Nadu	[21]
	Antimicrobial effect, anti-inflammatory activity	Leaf	Maceration	India	Dharmapuri, Tamil Nadu	[64]
	Fever, neuralgia, urinary infection, rheumatism, inflammation, liver and kidney diseases	Leaf	Maceration and decoction	India	Varakkalpattu village, Cuddalore District, Tamil Nadu	[51]
*M. mammosa*	Breast cancer	Whole plant	Maceration	Indonesia	Surabaya	[65]
	Diabetic therapy	Leaf	Infusion	Indonesia	Meru Betiri National Park, Jember	[56]
	Diabetic ulcers	Whole plant	Maceration	Indonesia	Klaten, Central Java Province	[57]
*M. peltata*	Anti-inflammatory, analgesic, anti-cancer, anti-viral, anti-malarial, anti-bacterial, and anti-fungal	Whole plant	Maceration	Philippines	Rogongon, Iligan City	[19]
	Anti-cancer, diarrhea, abdominal pain, cough, sore eyes, wound and inflammation	Leaf	Maceration and Fractionation	Indonesia	Padang City, West Sumatra	[58]
*M. tridentata*	Rheumatism, hemiplegia, piles, swellings, and urinary disorders	Root	Maceration	India	Udupi, Manipal	[53]
	Toothache	Whole plant	Maceration	India	Xavier’s College campus, Palayamkottai, Tirunelveli District, Tamil Nadu	[55]
	Astringent, calefacient, laxative, anodyne, hemiplegia, hemorrhoids, uropathy, mouth wash, piles, inflammation, fever, and leprosy	Aerial parts	Maceration	India	Tamil Nadu Medicinal Plant Farms and Herbal Medicine, Chennai	[66]
	Piles, swellings, rheumatism, stiffness of the joints, hemiplegia, and urinary infections	Whole plant	Maceration	India	Coimbatore, Tamil Nadu	[54]
	Treatment of diabetes	Root	Maceration	India	Coimbatore, Tamil Nadu	[52]
*M. umbellata*	Antibacterial, antifungal, and anti-inflammatory	Leaf	Maceration	Colombia	Pueblo Nuevo, Bolívar,	[60]
*M. yunnanensis*	Typhoid and stroke treatment	Whole plant	Decoction	China	Heqing Country, Dali Prefecture, Yunnan province	[60]
	Stroke hemiplegia, typhoid fever, and headache	Fruit/Leaf	Infusion	China	Heqing County of Yunnan Province	[61]
*M. vitifolia*	Fever, headache, eye inflammation, rheumatism, dysentery, jaundice, and urinary diseases	Leaf	Maceration	Bangladesh	Chittagong	[20]

## Supplementary Materials

The following are available online at https://www.mdpi.com/article/10.3390/plants10102070/s1, Table S1: The full distribution listing of *Merremia* species around the world.

## Abbreviations

A549	Lung Carcinoma
ABTS	2,2′-azino-bis (3-ethylbenzothiazoline-6-sulfonic acid)
ACE-2	Angiotensin-Converting Enzyme 2
ACEI	Angiotensin-Converting-Enzyme Inhibitory
AGS	Stomach Cancer Cells
APFME	Aqueous Portion of Fractionated Methanol Extract
BHA	Butylated Hydroxyanisole
BW	Body Weight
CAFDs	Caffeic Acid Derivatives
COLO 320	Colon Cancer Cells
DM	Dry Matter
DMSO	Dimethyl sulfoxide
DPPH	1,1-diphenyl-2-picrylhydrazyl
DU-145	Prostate Carcinoma
EDTA	Ethylenediaminetetraacetic Acid
ESRD	End-Stage Renal Disease
EGFR	Epidermal Growth Factor Receptor
ELISA	Enzyme-Linked Immunosorbent Assay
FITC	Fluorescein Isothiocyanate-Conjugated
FRAP	Ferric Reducing Antioxidant Power
GAE	Gallic Acid Equivalents
HPMC	Hydroxypropylmethylcellulose
IP	Intraperitoneally
KB	Mouth Carcinoma
LPS	Lipopolysaccharide
MCF-7	Breast Cancer Cells
MIA-PaCa-2	Pancreas Carcinoma
MIC	Minimum Inhibitory Concentration
MTT	3-(4,5-Dimethylthiazol-2-yl)-2,5-diphenyltetrazolium bromide
Na CMC	Sodium Carboxymethylcellulose
OH•	Hydroxyl Radicals
PI	Propidium Iodide
PO	Per os
RP-HPLC	Reverse Phase-High Performance Liquid Chromatography
TBARS	Thiobarbituric Acid Reactive Substances
TGA-capped-CdTe QDs	Thioglycolic Acid-Capped Cadmium Telluride Quantum Dots
TNF-α	Tumour Necrosis Factor Alpha
UPLC-MS/MS	Ultra Performance Liquid Chromatography-Tandem Mass Spectrometry
USDA	United State Department of Agriculture
VERO	Monkey Normal Kidney Epithelial Cells
WFO	World Flora Online
WHO	World Health Organization
WoS	Web of Science
