# Supplementary material for: Research Progression of the Genus Merremia: A Comprehensive Review on the Nutritional Value, Ethnomedicinal Uses, Phytochemistry, Pharmacology, and Toxicity
Source: Plants (Basel). 2021 Sep 30;10(10):2070. doi: 10.3390/plants10102070 (PMC8537340; doi:10.3390/plants10102070)
Supplement: Supplementary file 1 [file plants-10-02070-s001.zip › plants-1362824-supplementary.pdf]

Table S1. The full distribution listing of *Merremia* species around the world.

Angola
Australia
Benin
Bangladesh
Botswana
Brazil North
Brazil Southeast
Burundi
Burkina Faso
Cameroon
Cambodia
Chad
Central African Republic
China North-Central
China South-Central
China Southeast
Costa Rica
Congo
Cuba
Dominican Republic
Ethiopia
Fiji
France
Gambia
Gabon
Guatemala
Ghana
Guinea
Guyana
Guinea-Bissau
Haiti
Indonesia
Ivory Coast
India
Japan
Kiribati
Kenya
Lesotho
Laos
Liberia
Malawi
Madagascar
Mali
Mauritius
Mauritania
Malaysia
Mexico Gulf
Mexico Central
Mexico Northeast
Mexico Southeast

Mexico Southwest
Myanmar
Mozambique
Mongolia
Nepal
Namibia
Nicaragua
New Guinea
Niger
Nigeria
Panamá
Pakistan
Philippines
Russia
Rwanda Sierra Leone
Senegal
Solomon Island
South Africa
Sri Lanka
Somalia
Sudan
Suriname
Swaziland
Tanzania
Taiwan
Thailand
Togo
Uganda
Vietnam
Venezuela
Western Australia
Zaïre
Zambia
Zimbabwe
